# Associations between sociodemographic characteristics and knowledge about antibiotics and antibiotic resistance and usage of antibiotics from a One Health perspective in rural Bangladesh: a descriptive cross-sectional study

**DOI:** 10.1136/bmjopen-2025-104131

**Published:** 2025-12-18

**Authors:** Joseph Paul Hicks, Rumana Huque, Fariza Fieroze, Md Badruddin Saify, Tim Ensor, Khaleda Islam, Sophia Latham, Jessica Mitchell, Aninda Rahman, Md Nazmul Islam, Rebecca King

**Affiliations:** 1 Nuffield Centre for International Health and Development, University of Leeds, Leeds, UK; 2 ARK Foundation, Dhaka, Bangladesh; 3 Department of Livestock and One Health, Institute of Veterinary and Ecological Sciences, University of Liverpool, Liverpool, UK; 4 College of Medicine and Veterinary Medicine, The University of Edinburgh Global Academy of Agriculture and Food Systems, Edinburgh, UK; 5 Directorate General Health Services, Government of the People’s Republic of Bangladesh Ministry of Health and Family Welfare, Dhaka, Bangladesh

**Keywords:** Community Participation, PUBLIC HEALTH, Antibiotics

## Abstract

**Abstract:**

**Objectives:**

We explored how key sociodemographic characteristics were associated with correct knowledge about antibiotics and antibiotic resistance (ABR) and appropriate usage of antibiotics from a One Health perspective among rural community members in Bangladesh.

**Design:**

Cross-sectional single-period survey.

**Setting:**

Rural villages in Cumilla district, Bangladesh.

**Participants:**

Eligibility criteria: aged ≥18. Across 50 clusters of villages, we approached 2160 community members and 2187 (98.8%) agreed to participate.

**Primary and secondary outcome measures:**

Primary outcomes: we collected two knowledge outcomes measuring the number of correctly answered binary/multiple-choice questions about (1) antibiotics and ABR and appropriate usage of antibiotics in relation to human illness and (2) antibiotics and ABR and appropriate usage of antibiotics in relation to animal health and the environment. Secondary outcomes: self-reported awareness of (1) antibiotics and (2) ABR.

**Results:**

Several sociodemographic characteristics were associated with variation in both knowledge outcomes. Education showed the strongest associations, with higher education levels associated with higher knowledge scores. For example, compared with having no formal/incomplete primary education, having higher education was associated with 10 percentage points (95% CI 8 to 12) and 6 percentage points (95% CI 3 to 8) higher mean knowledge scores for the knowledge outcomes 1 and 2, respectively. Having worked in the last month compared with not having worked was also weakly positively associated with both knowledge outcomes, and being female compared with being male was also weakly negatively associated with both knowledge outcomes.

**Conclusions:**

Better public education is required to tackle ABR in Bangladesh but correct knowledge about antibiotics and ABR and appropriate usage of antibiotics in relation to humans, animals and the environment varies in relation to individuals’ education level, sex and working status. To maximise their effectiveness, interventions to tackle ABR must be flexible given recipients’ sociodemographic characteristics and pre-existing knowledge levels.

**STRENGTHS AND LIMITATIONS OF THIS STUDY**

- Our study achieved a very high response rate (98.8%) which greatly minimised the risk of any selection bias.
- We did not use a probability sampling approach, but we did use a pragmatic cluster-sampling approach that was explicitly designed to maximise generalisability and representativeness of community samples when resources are limited.
- We looked at correct knowledge about antibiotics and antibiotic resistance and appropriate usage of antibiotics from a holistic, One Health perspective, but we had to rely on self-reported responses and we were not able to look at any actual practices in relation to these issues due to logistical constraints.

## Introduction

 Antibiotic resistance (ABR), and more generally antimicrobial resistance (AMR), is considered a major global health problem that, due to the complex and diverse nature of their drivers, is best addressed through a One Health approach.^1^ The use and misuse of antimicrobials for human and animal health is considered to be the main driver behind the global increase in AMR.^2^ Among the world’s major geographical regions, South Asian countries have the second highest average risk of death due to an antimicrobial resistant infection after sub-Saharan African countries.^3^ The level of public awareness and correct knowledge about the appropriate use of antimicrobials also appears to be low across all WHO regions,^4^ and research in low- and middle-income countries (LMICs) also suggests that patients commonly pressure clinicians or pharmacists to inappropriately prescribe or dispense antimicrobials (eg, references^5 6^). It has therefore been recommended by the WHO^7^ and various national^8^,^10^ and transnational governments^11^ that improving public knowledge about antimicrobials, and particularly antibiotics as the most frequently used class of antimicrobial,^12^ should be a key priority.

In Bangladesh, like in most South Asian countries, antimicrobials are formally restricted as prescription-only medicines, but in practice, they are widely and freely purchased in pharmacies and from informal drug sellers, often without appropriate medical advice or even medication packaging.^13 14^ Previous studies in Bangladesh have also found a broad lack of awareness in community settings about the nature of antimicrobials and how to use them appropriately for human and animal health issues and animal husbandry,^14^,^16^ as well as limited levels of correct knowledge about antibiotics and ABR.^17^,^19^ However, these studies have focused on urban populations,^17^ individuals seeking health services,^18^ or have targeted the general population but using very biased, purposive sampling methods (online surveys),^19^ while other studies from this context have focused on narrow groups such as students (eg, reference^20^).

Studies from other LMICs focusing on the public have found important associations between sociodemographic characteristics, such as education levels and sex, and levels of knowledge about antibiotics and ABR (eg, references^21^,^23^). These findings can help inform the targeting and focus of community-focused public health efforts aimed at tackling antibiotic/AMR (such as educational and engagement interventions). Therefore, here we aim to understand what associations exist between key sociodemographic characteristics and levels of correct knowledge and awareness relating to antibiotics and ABR and appropriate antibiotic usage from a One Health perspective, within the general rural Bangladeshi population.

In rural Bangladesh, we are currently evaluating the effectiveness of a community-led engagement programme aimed at improving awareness and correct knowledge about antibiotics and ABR and appropriate usage of antibiotics from a One Health perspective via a cluster randomised controlled trial. The 12-month-long community engagement programme, known as the community dialogue approach, addresses many issues related to antimicrobials and AMR from a One Health perspective, focusing on antimicrobials and AMR in relation to humans, animals and the environment. For our trial, we have conducted a (pre-intervention) baseline household survey in Cumilla district, Bangladesh. The eventual primary use of this baseline data will be to increase precision and reduce bias in our effectiveness analyses that will use our (post-intervention) endline data.

However, for this paper, we are using the trial baseline data as if it was a stand-alone cross-sectional survey, and we are analysing it to address three broad descriptive research questions^24^ following our overall aim, with the third research question being our primary focus. First, among adult (≥18) rural community members in Cumilla district in 2022, what percentage reported being aware of the existence of antibiotics as a type of medicine and how does this vary across key sociodemographic characteristics? Second, restricting this target population to just those who report awareness of antibiotics, what percentage report being aware of the existence of ABR (we do not look at how this outcome varies across sociodemographic characteristics given the similarity to the previous outcome)? Third, within this same target population (ie, just among those who report awareness of antibiotics) what is the level of correct knowledge about antibiotics and ABR and appropriate usage of antibiotics from a One Health perspective (in relation to human health conditions, animal health and the environment) and how does this vary across key sociodemographic characteristics? The goal in answering these questions is to inform policy and practice in Bangladesh and similar contexts in relation to which sociodemographic groups require most attention and resources, particularly regarding future interventions targeting community awareness and knowledge on these issues.

## Methods

Methodological details on the setting, clusters, cluster and participant sampling, outcomes (including all questions forming the knowledge tests) and statistical analyses are provided in the supplementary materials.

### Approach and reporting

We followed the recent framework for descriptive epidemiology^24^ and other recent guidance on descriptive epidemiology^25^ when planning, analysing and reporting the study, and included their ‘Items That Should Be Included in Reports of Descriptive Studies’ (see table 1 in reference 24) as a checklist in the supplementary materials. We also report according to the strengthening the reporting of observational studies in epidemiology checklist for observational cross-sectional studies.^26^

### Study design

Cross-sectional single-period survey (based on data from the baseline survey of a cluster trial).

### Setting

In Bangladesh, there are 64 districts and 495 subdistricts. For this trial, we carried out all work in Cumilla district in south-eastern Bangladesh, around 100 km from Dhaka, where during sampling there were 16 subdistricts and 3687 villages. Among these 16 subdistricts, we purposively selected five predominantly rural subdistricts for the trial that were broadly representative of the distribution of literacy levels (as a proxy for education levels) among Cumilla’s rural subdistricts (online supplemental table S1), and which we also had previous experience of working in.

### Participants

Survey participants were aged 18 years or above and must have lived within their village for the 12 months prior to the survey without having lived elsewhere for more than a month.

### Clusters

Our clusters are the populations living in the villages within the catchment areas of community clinics (CCs), which are rural, basic, primary care facilities. Each CC usually covers 4–6 (but sometimes >10) villages and around 6000 individuals.

### Sample size

Our trial’s sample size calculations resulted in an aim to sample a total of 50 clusters and 44 individuals within each cluster for a total of 2200 individuals.

### Cluster and participant sampling

From the 144 CCs within our five chosen subdistricts, we randomly selected 10 such that within each subdistrict all selected CCs were ≥2.5 km apart (based on linear distances). Each CC and the villages it served then formed our clusters. For the household survey, we used a pragmatic, multistage sampling approach to select participants from clusters, following the older WHO Expanded Programme of Immunisation cluster sampling ‘spin the pen’ method. The is not a true random probability sampling method, but it aims to generate broadly representative samples while avoiding the more serious sampling bias caused by most purposive approaches in a simple, cost-effective way. Briefly, within each cluster, our data collectors aimed to sample 22 participants from the village that the cluster’s CC was located within, and a further 22 participants from the nearest adjacent village in the same cluster (or if there was just one village in the cluster, then they would sample all 44 participants from that village). From each selected household, our data collectors asked the first available eligible female or male to participate to maintain a 1:1 female:male sampling ratio. See online supplemental materials ‘Cluster sampling and participant sampling’ section for more details.

### Data collection, questionnaire and outcomes

We collected data for the trial baseline survey via a digital questionnaire (written and delivered verbally in Bangla). For this survey study, though, we just analyse four outcomes derived from questions in this larger questionnaire. Outcome 1: correct knowledge about antibiotics and ABR and appropriate usage of antibiotics in relation to human health. Individuals’ knowledge about these issues is measured as a test score based on the sum of correct answers to 25 questions on these topics, which require a true/false, appropriate/inappropriate, trust/do not trust type response, where only one of the responses is either judged to be factually correct or in-line with appropriate practice. Outcome 2: correct knowledge about antibiotics and ABR and appropriate usage of antibiotics in relation to animal health and the environment. Individuals’ knowledge about these issues is similarly measured as a test score based on the sum of correct answers to 20 questions on these topics, which again require the same type of responses and are judged in the same way as the other knowledge outcome. Individuals who reported being unaware of antibiotics automatically received a score of 0 on each test score.

The questions used to derive the knowledge scores were based on the specific information that was planned to be provided during the community dialogue sessions, and therefore community members who either attended the community dialogues or discussed them with other members of the community who attended them should be aware of this information (at endline in the trial) if the intervention was effective. The content of the questions and therefore the relevant content of the community dialogues was primarily based on information and guidance from the WHO, the Food and Agriculture Organisation, and the World Organisation for Animal Health (OIE) (2, 3), as well as the content of previous related surveys (from other research groups) that have also aimed to assess this type of knowledge among community members (eg, (4–7)), our earlier qualitative exploratory work and small-scale quantitative survey work in this context (8, 9), which guided the development of the community dialogues approach, and the relevant expertise of our team members.

Outcome 3: self-reported awareness of antibiotics: a binary outcome of whether an individual responded ‘yes’ versus ‘no’ or ‘don’t know’ to the question ‘Have you ever heard of a type of medicine known as an antibiotic or antibiotics?’ Outcome 4: self-reported awareness of ABR: a binary outcome of whether an individual responded ‘yes’ versus ‘no’ or ‘don’t know’ to the question ‘Have you ever heard of any of the terms ‘ABR’, ‘AMR’ or ‘drug resistance?’

### Target population

Our target population for our first two research questions and analyses related to awareness of antibiotics and ABR is all adult (≥18) rural community members in Cumilla district in 2022. For our third research question and analyses related to correct knowledge about antibiotics and ABR and appropriate usage of antibiotics from a One Health perspective (our test scores) in 2022, the target population is the subset of this target population who report awareness of antibiotics (and are thereby able to answer knowledge questions about antibiotics and ABR).

### Statistical analyses

We used R statistical software^27^ for all analyses.

#### Participant characteristics

We describe the sociodemographic characteristics of the sampled participants using standard summary statistics.

#### Inferential analyses

Full details of all inferential analyses are given in the supplementary materials (see the section ‘Statistical analyses: additional details’) and we just provide an overview here. As this was a descriptive study (and as per our research questions), we aimed to estimate the likely value of the targeted descriptive estimands as they actually exist in the target populations of interest, primarily to help inform planning and policy in relation to identifying which sociodemographic groups require most attention from public-facing interventions addressing AMR. We therefore did not adjust or standardise the point estimates in relation to any covariates apart from the sampling strata (see below in this section).^24 25^ Consequently, all results closely reflect the observed data and the point estimates that could be obtained ‘by hand’, but we used a consistent, model-based approach to estimate all estimands (see ‘Descriptive estimands’ in the supplementary materials for full details of the estimands) as this allowed us to estimate percentages and percentage point differences (rather than the more common odds-ratio scale contrasts)^28^ along with their 95% CIs.

We estimated two main sets of results. First, we summarised all our outcomes as percentages for both the overall target population of interest and for subgroups within that target population, as defined by key sociodemographic characteristics (eg, sex and age groups—in practice, these were all categorical variables). Second, we then looked at unadjusted associations between the outcomes and variation in each of the key sociodemographic characteristics by estimating, for each characteristic, the percentage point difference for each outcome between a natural reference group for that characteristic and all other groups (eg, for sex: the female vs male percentage point difference).

To compute these results, we used marginal effects (sometimes instead called standardisation) based methods.^29^ This involved using suitable generalised linear models (GLMs): either with Bernoulli distributions and logit links for our two binary outcomes (the self-reported awareness indicators) or binomial distributions and log links for our two knowledge scores (treating them as binomial outcomes with each participant having n correct answers out of N trials (ie, the number of questions)). To estimate the overall observed percentage for each outcome and its 95% CI, we fitted suitable GLMs with just one covariate for the sampling strata (subdistrict), which was included only to increase precision^30^ (therefore we do not present association results for subdistrict as they do not relate to our research questions and the subdistricts are considered broadly interchangeable with any other set of subdistricts from Cumilla). We then used the R package *marginaleffects*^31^ to compute the observed percentages and associated 95% CIs from each model. To estimate the observed percentage for each outcome within each sociodemographic subgroup and their associated 95% CIs, we created separate GLMs for each outcome-sociodemographic combination with each GLM including just two covariates: one for the sociodemographic group of interest and one for the sampling strata (again just to increase precision). We again then used marginal effects methods to compute the observed subgroup-specific outcome percentages and associated 95% CIs from each model. Finally, we then used marginal effects methods on these same models to compute the observed percentage point differences and associated 95% CIs when comparing each outcome between the subgroups of each sociodemographic variable.

For our research questions comparing our outcomes between age groups and education levels, we also computed additional comparisons with the opposite subgroup set as the reference subgroup compared with the main results. For space, we present these in the supplementary materials (see ‘Additional comparisons between age and education level subgroups’).

For all estimands, we computed 95% CIs based on the delta method and using ‘HC3’ cluster robust SEs to adjust the SEs for the clustered sampling design (see online supplemental table S2 footnote 4 for justification), with clustering at this highest level also adjusting for any clustering of knowledge score questions within participants when analysing the knowledge scores as binomial outcomes.^32^ We base all our statistical inferences in relation to the relevant target population on these 95% CIs. For all analyses, there were no missing data other than from sampled individuals who did not consent to participate (and who were therefore not included in the analyses).

### Patient and public involvement

In the context of the wider project that this study is a part of, the public were first involved at the initial feasibility and exploratory study stage. The overall aim of the wider project is to develop and evaluate a community engagement intervention, with the intervention being delivered by public volunteers from the communities where the intervention is being delivered. Therefore, interested individuals from the communities where the study was to be carried out were involved at the initial feasibility and exploratory study stage via face-to-face discussions with members of the in-country research team during visits to those communities.

The study design, focus of the research questions and broad nature of the outcome measures were not planned with patient or public involvement. However, as part of the wider project that this study is a part of, individuals from the study communities were involved in discussions around the acceptability and feasibility of the project (including feedback related to the burden and time requirements for participants) and in testing the clarity and acceptability of the questions that were used to create the outcomes via pilot testing. There was no direct public involvement in the recruitment to the survey; however, for the wider project, members of the study communities were involved in spreading the word about the community engagement intervention within their communities, thereby helping recruit individuals to deliver the intervention.

Members of the study communities, particularly those involved with delivering the intervention as part of the wider project, will be involved in meetings in the study communities where members of the in-country research team will provide feedback about the results from the wider project (as well as the results from this study).

## Results

### Cluster size, number of participants and participants’ characteristics

Between 21 September 2022 and 24 November 2022, we approached 2187 participants across 50 clusters and invited them to participate in the survey. A total of 2160 (98.8%) participants consented and completed the survey. We present cluster sizes and participant characteristics in table 1.

**Table 1:** Cluster size and participants’ characteristics

N	2160
Cluster size	44.0 (40.0, 44.0)
Sex	
Male	50.2% (1084)
Female	49.8% (1076)
Age	
18–25	24% (510)
26–32	18% (392)
32–40	21% (457)
41–55	21% (452)
56+	16% (349)
Religion	
Muslim	96% (2080)
Hindu	4% (80)
Education	
No formal education/incomplete	13% (290)
Incomplete primary	14% (307)
Primary	9% (193)
Incomplete secondary	31% (676)
Secondary or incomplete higher	18% (381)
Higher	15% (313)
Worked in the past 30 days?	
Yes	60% (1297)
No	40% (863)
Type of work	
Manual	45% (389)
Business	26% (226)
Professional	14% (121)
Other	15% (127)
Does the household own any animals?*	
Yes	75% (1617)
No	25% (543)
No of individuals living in household	
1–4	39% (839)
5–6	40% (862)
7+	21% (459)

Data are median (range) and % (frequency).

* Full question: ‘Do you or your family currently own any poultry, cattle, goats, sheep or fish for producing food, or to eat them, or to sell or trade?’

### Levels of awareness of antibiotics and antibiotic resistance

82.1% (95% CI 79.2% to 85%; n=1774/2160) of all participants responded reported having ‘… heard of a type of medicine known as an antibiotic or antibiotics’, while 12% (95% CI 10.2% to 13.8%, n=213/2160) of all participants reported having ‘heard of any of the terms “antibiotic resistance”, “AMR” or “drug resistance”?’

### Association between sociodemographic characteristics and self-reported awareness of antibiotics

There were several clear associations between variation in sociodemographic characteristics and levels of reported awareness of antibiotics (table 2). The strongest involved education, where the percentage of reported awareness of antibiotics among individuals who had higher education was 31 percentage points (95% CI 27 to 36) higher compared with the percentage among individuals with no formal education. Having successively lower levels of education (compared with the same reference level) was all associated with smaller but still clear associations, and there was a clear, moderately higher level of reported awareness among those with higher education compared with those with primary/incomplete secondary but not in comparison to those with secondary/incomplete primary (see online supplemental table S3 in the supplementary materials). Age was also strongly negatively associated with awareness, with the percentage of reported awareness of antibiotics among individuals aged 56+ being −17 percentage points (95% CI −23 to –11) lower compared with the percentage among individuals aged 18–25. Successively younger age groups (compared with the same reference level) were all associated with smaller but still clear associations, aside from those aged 26–32, and there was a clear, moderately lower level of reported awareness among those aged 56+ compared with to those aged 26–32 or compared with those aged 33–40 but not in comparison to those aged 41–55 (see online supplemental table S3 in the supplementary materials). There were also clear but smaller associations between differences in sex and work status, with the percentage of reported awareness of antibiotics among females being −5 percentage points (95% CI −8 to –2) lower than among males, and the percentage of reported awareness of antibiotics among those reporting having worked within the past 30 days being 4 percentage points (95% CI 1 to 7) higher than among those reporting not having worked within the past 30 days.

**Table 2:** Percentage self-reported awareness of antibiotics by sociodemographic group and between-group differences

Characteristic and subgroups	% self-reported awareness of antibiotics (95% CI)	Frequency	Percentage point difference compared with reference group (95% CI)
Sex			
Male	84.5% (81.2 to 87.8)	916/1084	Reference
Female	79.8% (76.3 to 83.2)	858/1076	−4.7 (−7.6 to 1.9)
Age			
18–25	89.5% (85.9 to 93.2)	457/510	Reference
26–32	86.5% (83 to 89.9)	339/392	−3 (−6.5 to 0.4)
33–40	83.2% (78.2 to 88.1)	380/457	−6.4 (−11.5 to 1.3)
41–55	76.3% (71.3 to 81.3)	344/452	−13.2 (−18.6 to 7.8)
56+	72.7% (66.8 to 78.6)	254/349	−16.8 (−23.1 to 10.5)
Education level			
No formal education or incomplete primary	64.7% (59.8 to 69.7)	380/597	Reference
Primary or incomplete secondary	84.8% (81.8 to 87.8)	736/869	20.1 (15.1 to 25.1)
Secondary or incomplete higher	93.1% (90.3 to 96)	356/381	28.4 (23 to 33.8)
Higher	96.2% (93.4 to 99)	302/313	31.4 (26.8 to 36.1)
Worked in the past 30 days?			
Yes	84.4% (80.6 to 88.2)	729/863	3.8 (0.7 to 6.8)
No	80.6% (77.4 to 83.8)	1045/1297	Reference
Does the household own any animals?*			
Yes	82.4% (79 to 85.8)	1338/1617	1 (−3.8 to 5.8)
No	81.4% (76.9 to 85.9)	436/543	Reference

Sample size=2160 for each analysis of each sociodemographic characteristic. This included all individuals who agreed to participate in the survey out of the 2187 who were approached (response rate=98.8%). Outcome variable=a response of ‘Yes’ (1) or ‘No/don’t know’ (0) to the question ‘Have you ever heard of a type of medicine known as an antibiotic or antibiotics?’

* Full question: ‘Do you or your family currently own any poultry, cattle, goats, sheep or fish for producing food, or to eat them, or to sell or trade?’

### Overall levels of correct knowledge about antibiotics and ABR and appropriate usage of antibiotics from a One Health perspective

The overall mean percentage score for correct knowledge about antibiotics and ABR and appropriate usage of antibiotics in relation to human health was 42.6% (95% CI 41.8% to 43.6%), and the overall mean percentage score for correct knowledge about antibiotics and ABR and appropriate usage of antibiotics in relation to animal health and the environment was 69% (95% CI 67.4% to 70.6%).

### Association between sociodemographic characteristics and correct knowledge about antibiotics and ABR and appropriate usage of antibiotics from a One Health perspective

Among individuals who reported being aware of antibiotics, there were several clear associations between variation in sociodemographic characteristics and the mean test score for knowledge about antibiotics and ABR and appropriate usage of antibiotics in relation to human health (table 3). Again, the strongest involved education, where the mean percentage score among individuals who had higher education was 10 percentage points (95% CI 8 to 12) higher compared with the percentage among individuals with no formal education. Successively lower levels of education (compared with the same reference level) were all associated with smaller but still clear lower mean percentage scores, and there was a clear, moderately higher mean percentage score for those with higher education compared with those with primary/incomplete secondary or compared with those with secondary/incomplete primary (see online supplemental table S4 in the supplementary materials). However, unlike for self-reported awareness of antibiotics, there were no clear associations with age group. There were also clear but smaller associations between differences in sex and work status, with the percentage score among females being −2 percentage points (95% CI −3 to –1) lower than among males, and the percentage score among those reporting having worked within the past 30 days being 2 percentage points (95% CI 1 to 4) higher than among those reporting not having worked within the past 30 days.

**Table 3:** Sociodemographic variation in the level of correct knowledge about antibiotics and antibiotic resistance and appropriate usage of antibiotics in relation to human health (among individuals reporting awareness of antibiotics)

Characteristic and subgroups	Mean test score (% (95% CI))	Percentage point difference compared with reference group (95% CI)
Sex		
Female	41.6% (40.6 to 42.6)	−2 (−3.2 to 0.8)
Male	43.6% (42.5 to 44.8)	Reference
Age		
18–25	43% (42 to 44.1)	Reference
26–32	42.9% (41.1 to 44.6)	−0.2 (−2 to 1.6)
33–40	42.3% (40.8 to 43.8)	−0.8 (−2.3 to 0.8)
41–55	42.8% (41.4 to 44.3)	−0.2 (−1.8 to 1.3)
56+	42.1% (40.2 to 44)	−1 (−3.1 to 1.2)
Education level		
No formal education or incomplete primary	39.5% (37.9 to 41.2)	Reference
Primary or incomplete secondary	40.7% (39.6 to 41.8)	1.2 (−0.3 to 2.7)
Secondary or incomplete higher	44.2% (42.8 to 45.5)	4.6 (2.8 to 6.5)
Higher	49.6% (48 to 51.2)	10.1 (7.9 to 12.2)
Worked in the past 30 days?		Reference
Yes	44% (42.8 to 45.3)	2.4 (1.2 to 3.6)
No	41.7% (40.8 to 42.6)	Reference
Does the household own any animals?*		
Yes	42.6% (41.6 to 43.6)	−0.4 (−1.6 to 0.8)
No	43% (41.7 to 44.3)	Reference

Sample size=1774 for each analysis of each sociodemographic characteristic. This included all individuals who agreed to participate in the survey and reported being aware of antibiotics (82.1% of the 2160 individuals who were approached and agreed to participate in the survey). Knowledge test comprised 25 binary/multiple-choice questions assessing individuals’ correct knowledge about antibiotics and ABR and appropriate usage of antibiotics in relation to human health. Each question response was assessed as either correct and appropriate or incorrect/inappropriate, with the overall test score (ie, outcome variable values) being a simple sum of the number of correct and appropriate responses.

* Full question: ‘Do you or your family currently own any poultry, cattle, goats, sheep or fish for producing food, or to eat them, or to sell or trade?’

Among individuals who reported being aware of antibiotics, there were also several clear associations between variation in sociodemographic characteristics and the mean test score for knowledge about antibiotics and ABR and appropriate usage of antibiotics in relation to animal health and the environment (table 4). Again, the strongest involved education, where the mean percentage score among individuals who had higher education was 6 percentage points (95% CI: 3 to 8) higher compared with the percentage among individuals with no formal education. However, only secondary/incomplete higher education, but not primary/incomplete secondary education, was clearly associated with a higher mean test score compared with having no formal/incomplete primary education, and there was only a clear, slightly higher mean percentage score for those with higher education compared with those with primary/incomplete secondary, but not compared with those with secondary/incomplete primary (see online supplemental table S5 in the supplementary materials). Similar to the other knowledge test, there was again no clear variation due to age group, but there were clear, smaller associations between differences in sex and work status. Specifically, the percentage score among females was −4 percentage points (95% CI −6 to –3) lower than among males, and the percentage score among those reporting having worked within the past 30 days was 5 percentage points (95% CI 3 to 6) higher than among those reporting not having worked within the past 30 days.

**Table 4:** Sociodemographic variation in the level of correct knowledge about antibiotics and antibiotic resistance and appropriate usage of antibiotics in relation to animal health and the environment (among individuals reporting awareness of antibiotics)

Characteristic and subgroups	Mean test score (% (95% CI))	Percentage point difference compared with reference group (95% CI)
Sex		
Female	66.9% (65.1 to 68.6)	−4 (−5.5 to 3)
Male	71% (69.2 to 72.8)	Reference
Age		
18–25	69.9% (68 to 71.7)	Reference
26–32	68.3% (65.8 to 70.9)	−1.5 (−3.4 to 0.4)
33–40	68.6% (66.4 to 70.9)	−1.3 (−3.3 to 0.8)
41–55	70.1% (68.2 to 71.9)	0.2 (−1.5 to 1.8)
56+	67.5% (64.9 to 70.1)	−2.4 (−5.1 to 0.3)
Education level		
No formal education or incomplete primary	67% (64.6 to 69.5)	Reference
Primary or incomplete secondary	67.7% (65.8 to 69.6)	0.6 (−1.4 to 2.7)
Secondary or incomplete higher	70.6% (68.7 to 72.6)	3.6 (0.8 to 6.3)
Higher	72.8% (70.6 to 75)	5.7 (3.3 to 8.2)
Worked in the past 30 days?		
Yes	71.7% (69.9 to 73.5)	4.5 (3 to 6)
No	67.1% (65.5 to 68.8)	Reference
Does the household own any animals?*		
Yes	69.3% (67.6 to 71)	1 (−0.5 to 3)
No	68.2% (66.1 to 70.3)	Reference

Sample size=1774 for each analysis of each sociodemographic characteristic. This included all individuals who agreed to participate in the survey and reported being aware of antibiotics (82.1% of the 2160 individuals who were approached and agreed to participate in the survey). Knowledge test comprised 20 multiple-choice questions assessing individuals’ correct knowledge about antibiotics and ABR and appropriate usage of antibiotics in relation to animal health and the environment. Each question response was assessed as either correct and appropriate or incorrect/inappropriate, with the overall test score (ie, outcome variable values) being a simple sum of the number of correct and appropriate responses.

* Full question: ‘Do you or your family currently own any poultry, cattle, goats, sheep or fish for producing food, or to eat them, or to sell or trade?’

## Discussion

### Self-reported awareness of antibiotics and ABR

Levels of reported awareness of the existence of antibiotics as a type of medicine were high, but nearly one-fifth of individuals still reported they had not even heard of antibiotics, and this is likely to be an underestimate if you assume fear of admitting ignorance will have meant that some percentage of individuals will have reported awareness even if this was not really the case (ie, a social desirability bias, which is common bias in self-reported surveys).^33^ Conversely, levels of reported awareness of ABR, based on simple familiarity with common terms for the phenomena, were very low. In this setting, individuals are likely to encounter antibiotics due to their wide availability, and this may explain the relatively high level of antibiotic awareness. However, they may be much less likely to encounter information about ABR, which is both a complex concept and usually a hidden issue in LMICs, as most antibiotic resistant infections of humans and animals are not detected.^7 34^ Low levels of correct knowledge about antibiotics/antimicrobials/ABR/AMR have been found consistently in surveys in other LMICs, but the level of reported awareness about ABR appears to be particularly low in this setting compared with other LMICs.^21 22 35 36^ This clearly highlights the urgent need for better public education on these complicated issues within this setting.

### Association between sociodemographic characteristics and awareness of antibiotics and correct knowledge about antibiotics and ABR and appropriate usage of antibiotics from a One Health perspective

By far the largest source of sociodemographic variation in both awareness of antibiotics and levels of correct knowledge about antibiotics and ABR and appropriate usage of antibiotics from a One Health perspective was in relation to education levels, where higher levels of education (compared with no formal education/incomplete primary education) were increasingly strongly associated with increased levels of reported awareness of antibiotics and higher average scores on the two knowledge tests. Many other survey studies across many LMICs have also found associations between higher levels of education and increased levels of awareness, correct knowledge, correct attitudes and sometimes also appropriate reported practices in relation to antibiotics/antimicrobials and ABR/AMR.^2122 35^,^38^ While unsurprising to public health researchers, the ultimate causal factors behind these associations are arguably less clear. Formal education level may clearly be one causal factor, but it could also be a proxy for other causal factors driving the observed associations, such as innate intelligence or aspects of intelligence (eg, the capacity to understand and memorise information), the likelihood of encountering these concepts in your peer group, or interest in seeking out relevant information. However, these are speculations that would require further research to explore.

There were also increasingly negative associations between older age groups (compared with the youngest age group) and decreased levels of reported awareness of antibiotics, but interestingly there was no association between age and the two knowledge scores. Clearly, underlying factors correlated with age will be driving the associations as there is no plausible reason for age per se to affect awareness of antibiotics. Older age groups reported lower levels of education than younger age groups on average (data not shown), so the same underlying processes driving the education associations may also be at play here too. However, other factors correlated with age may well also play a role, such as increased access to relevant medical information via social media/internet among younger age groups leading to greater awareness and knowledge. Again, these are speculative suggestions though that would require further research to explore.

We also found a clear association between being female and having a slightly lower chance of being aware of antibiotics and on average slightly lower scores on both knowledge tests. In rural Bangladesh, as is common in most other LMICs, women are typically the primary caregivers for any children they may have, and often other relatives too, such as husbands and elderly relatives. Therefore, it would seem plausible that they would be more likely than men to encounter antibiotics and information about them when seeking healthcare for their children/other relatives. Again, sex-based differences in other correlated characteristics may be ultimately causing these associations, such as women having on average lower levels of education than men leading to those underlying causal factors associated with education coming into play. The modest amount of relevant survey-based literature from LMICs that has examined the association between sex and awareness/knowledge related to antibiotics/antimicrobials/ABR/AMR has shown very varied findings (eg, references^2122 35^,^37 39 40^), but this may just be due to varied (and questionable) statistical approaches, such as inappropriately adjusting away sex-based differences in ostensibly descriptive studies.^41^ Clearly, further research is needed on this important question.

In terms of the other sociodemographic characteristics examined, there was also a clear but small association between reporting having worked in the past 30 days (compared with not) and having a higher chance of being aware of antibiotics and on average higher scores on both knowledge tests. Again, this may well be due to underlying correlated factors, such as those working having higher education levels, but this would need further exploration.

### Strengths and limitations

We had a very high response rate, which should have helped reduce selection bias and improved generalisability. However, we did not use a probability sampling approach to select the study subdistricts, or the study clusters or participants, which may have led to selection bias and harmed the generalisability of the results. We did, however, choose the subdistricts to have a representative distribution of adult literacy levels (as a proxy for education) compared with all of Cumilla’s subdistricts, and we also used a participant sampling approach that, while not a probability sampling method, has been designed to pragmatically reduce the likelihood of obtaining a less representative sample in such surveys.

Our awareness outcomes relied on self-reported responses to simple questions about whether the respondent had heard about antibiotics/ABR (or similar terms). Therefore, there is clearly the likelihood of some information bias affecting these outcomes, particularly social desirability bias^33^ among respondents who had not actually previously heard of antibiotics and/or ABR falsely claiming to have heard of them due to a fear of being seen as ignorant. Our knowledge outcomes were also based on self-reported proxy measures of the underlying phenomena we ideally wanted to measure, that is, actual knowledge levels, which means there is a strong possibility of outcome measurement error due to misunderstandings, respondents guessing answers to the test questions, or simply dishonest responses, all of which can lead to bias or reduced precision in estimates.^33^

## Conclusions

Generalisable strategies for creating and implementing community education and engagement interventions to tackle health issues like AMR have been developed for use in this context and more widely in other LMIC contexts,^42^,^47^ and some studies have shown promising results within community and healthcare settings.^48^,^51^ However, the level of evidence on their effectiveness in this area is currently weak and mixed, with a particular lack of robust experimental evidence.^52^ The trial that this survey study is a part of will be specifically addressing this research question within this context.

Our findings here emphasise that such programmes must ensure that they are sufficiently flexible and can accommodate the wide variation in levels of awareness and understanding on these issues that exist, and that they should particularly target individuals with lower levels of education, women and older individuals, as these groups are likely to have the lowest levels of awareness and understanding of these issues. Other groups defined by other important sociodemographic factors, such as ethnicity, may of course also be important to consider in this way, particularly for other settings, and so context-dependent knowledge must clearly be used when developing such programmes. Further and ongoing survey work is also clearly required to better understand public awareness, understanding, attitudes and ultimately practices in relation to antibiotics and ABR and, more generally, antimicrobials and AMR, both within Bangladesh and in other LMICs.

## Supplementary material

10.1136/bmjopen-2025-104131 online supplemental file 1

## Data Availability

Data are available in a public, open access repository.
