# Supplementary material for: Associations between sociodemographic characteristics and knowledge about antibiotics and antibiotic resistance and usage of antibiotics from a One Health perspective in rural Bangladesh: a descriptive cross-sectional study
Source: BMJ Open. 2025 Dec 18;15(12):e104131. doi: 10.1136/bmjopen-2025-104131 (PMC12716581; doi:10.1136/bmjopen-2025-104131)
Supplement: online supplemental file 1 [file bmjopen-15-12-s001.docx]

Supplementary Materials for:

Associations between socio-demographic characteristics and knowledge about antibiotics and antibiotic resistance and usage of antibiotics from a One Health perspective in rural Bangladesh: a descriptive cross-sectional study

**Contents**

[1 Framework for Descriptive Epidemiology reporting checklist 3](#_Toc211335953)

[2 Strengthening the Reporting of Observational Studies in Epidemiology reporting checklist 7](#_Toc211335954)

[3 Descriptive estimands 12](#_Toc211335955)

[4 Methods: additional details 15](#_Toc211335956)

[4.1 Sample size 15](#_Toc211335957)

[4.2 Setting 15](#_Toc211335958)

[4.3 Clusters 16](#_Toc211335959)

[4.4 Cluster sampling 16](#_Toc211335960)

[4.5 Participant sampling 16](#_Toc211335961)

[4.6 Questionnaire 17](#_Toc211335962)

[4.6.1 Socio-demographic characteristics questions 18](#_Toc211335963)

[4.6.2 Awareness of antibiotics outcome question 20](#_Toc211335964)

[4.6.3 Awareness of antibiotic resistance outcome question 20](#_Toc211335965)

[4.6.4 Knowledge outcomes questions 20](#_Toc211335966)

[4.6.4.1 General and human-health-related knowledge questions on antibiotics and antibiotic resistance 20](#_Toc211335967)

[4.6.4.2 Animal-health-related knowledge questions on antibiotics and antibiotic resistance 23](#_Toc211335968)

[5 Statistical analyses: additional details 27](#_Toc211335969)

[5.1 Main analyses 27](#_Toc211335970)

[6 Additional comparisons between age and education level subgroups 31](#_Toc211335971)

[7 Author reflexivity statement 34](#_Toc211335972)

[8 References 37](#_Toc211335973)

# Framework for Descriptive Epidemiology reporting checklist

The below items were taken from Table 2 in “A Framework for Descriptive Epidemiology” (1) and converted into a reporting checklist.

| **Article Section and Item** | **Item No.** | **Recommendation(s)** | **Section** |
| --- | --- | --- | --- |
| Title and abstract | 1 | Explicitly state that this is a “descriptive study” in the title or the abstract. | Title and abstract |
|  | 2 | Summarize the target population and provide an informative and balanced summary of estimated disease occurrence in the abstract. | Abstract |
| **Introduction** |  |  |  |
| Background/rationale | 3 | State the motivation for the study, including, where relevant, the action that might be informed by the results. | Introduction |
| Objectives | 4 | State the descriptive estimand, explicitly including: | Introduction |
|  |  | (a) the target population (who would be affected by any decisions made as a result of the study?); | Introduction |
|  |  | (b) the health state to be summarized; | Introduction |
|  |  | (c) the measure of occurrence; and | Introduction |
|  |  | (d) any stratification variables, if applicable. | Introduction |
| **Methods** |  |  |  |
| Study design | 5 | (a) State whether the study is cross-sectional or longitudinal. | Methods: Study design |
|  |  | (b) Restate the measure of occurrence being targeted. | NA |
|  |  | (c) If the study is longitudinal, specify the time origin and follow-up period for the measure of occurrence; if the study is cross-sectional, specify the time anchor at which the health state is summarized for individuals. | NA |
| Setting | 6 | Describe any relevant features of the place and time in which the target population resides and across which data were collected. | Methods: Setting |
| Participants | 7 | (a) Describe the target population thoroughly in terms of person, place, and time. | Methods: Setting; Methods: Participants; Methods: Clusters; Methods: Target population |
|  |  | (b) Describe sampling into the study population (whether sampling was explicit or implicit, e.g., by inclusion in an administrative database); this includes eligibility criteria (see recommendations on data sources in item 10 below). | Methods: Participants; Methods: Clusters; Methods: Cluster and participant sampling |
|  |  | (c) Describe any restrictions on the analytical sample. | Methods: Target population |
| Outcome(s) | 8 | (a) State when and how the outcome is measured. | Methods: Data collection, questionnaire and outcomes |
|  |  | (b) Include estimates or discussion of the sensitivity and specificity of the study outcome definition relative to the gold standard. | NA |
|  |  | (c) List secondary outcomes or competing events of interest. | NA |
| Covariates | 9 | Specify any stratification or adjustment variables—clearly define how variables were collected or constructed. | Methods: Inferential analyses |
| Data sources/measurement | 10 | Clearly delineate any inclusion/exclusion criteria for membership in the data source, including the original purpose for which the data were collected, if not for the study at hand. | Methods: Participants; Methods: Clusters; Methods: Data collection, questionnaire and outcomes |
| Bias | 11 | Describe any assumptions or methods used to extrapolate data from the analytical sample to the study population and from the study population to the target population. | Methods: Target population; Methods: Inferential analyses |
| Statistical methods | 12 | (a) Describe the primary statistical methods used to estimate the measure of disease occurrence being targeted; discuss assumptions of that method in light of data limitations (e.g., assumption of independent censoring for people lost to follow-up). | Methods: Inferential analyses |
|  |  | (b) If any adjustment/standardization will be done, state the goal of such adjustment. | Methods: Inferential analyses |
| **Results** |  |  |  |
| Participants | 13 | Report numbers of individuals at each study stage (this is likely to be approximate for the target population); consider summarizing this information in a flow diagram. | Results: Cluster size, number of participants and participants’ characteristics |
| Descriptive data | 14 | (a) Report on the characteristics of the analytical sample in a “Table 1.” | Table 1 |
|  |  | (b) Indicate the number of participants with missing data for each variable used in the analysis. | See footnotes for Tables 2-4 and S3-5 |
|  |  | (c) If any weighting or imputation is done to reconstruct the study sample or target populations, include columns for those populations. | NA |
| Outcome data | 15 | (a) Present an overall (unstratified) estimate of the measure of occurrence of interest. | See Tables 2-4 |
|  |  | (b) Report “crude” (raw data in the analytical sample) and (if applicable) “corrected” (after any weighting or imputation) estimates. | See Tables 2-4 |
| Other analyses | 16 | Present prespecified stratum-specific or adjusted/standardized results. | See Tables 2-4 and S3-5 |
| **Discussion** |  |  |  |
| Key results | 17 | Summarize key results with reference to the study objectives. | Discussion: Reported awareness of antibiotics and antibiotic resistance; Discussion: Socio-demographic variation in awareness of antibiotics and levels of correct knowledge about antibiotics, antibiotic resistance, and the usage of antibiotics from a One Health perspective |
| Limitations | 18 | Summarize potential sources of selection bias and measurement error and any attempts to mitigate these biases. Discuss both the direction and magnitude of any potential bias. Integrating quantitative bias analysis into the study to guide these discussions is encouraged. | Discussion: Strengths and limitations section |
| Interpretation | 19 | (a) Avoid causal interpretations of descriptive results; avoid overinterpreting stratum-specific differences in measures of occurrence. | Results are interpreted descriptively |
|  |  | (b) Describe how results of this study might inform or improve public health or clinical practice. | Discussion: Reported awareness of antibiotics and antibiotic resistance; Discussion: Socio-demographic variation in awareness of antibiotics and levels of correct knowledge about antibiotics, antibiotic resistance, and the usage of antibiotics from a One Health perspective |
| See Am J Epidemiol. 2022 Jul 1;191(12):2063–2070. doi: [10.1093/aje/kwac115](https://doi.org/10.1093/aje/kwac115) | | | |

# Strengthening the Reporting of Observational Studies in Epidemiology reporting checklist

|  | Item No | Recommendation | Section |
| --- | --- | --- | --- |
| **Title and abstract** | 1 | (*a*) Indicate the study’s design with a commonly used term in the title or the abstract | Title |
|  |  | (*b*) Provide in the abstract an informative and balanced summary of what was done and what was found | Abstract |
| Introduction | | | |
| Background/rationale | 2 | Explain the scientific background and rationale for the investigation being reported | Introduction |
| Objectives | 3 | State specific objectives, including any prespecified hypotheses | Introduction |
| Methods | | | |
| Study design | 4 | Present key elements of study design early in the paper | Methods: Study design |
| Setting | 5 | Describe the setting, locations, and relevant dates, including periods of recruitment, exposure, follow-up, and data collection | Methods: Setting; Methods: Participants; Methods: Clusters; Results: Cluster size, number of participants and participants’ characteristics |
| Participants | 6 | (*a*) Give the eligibility criteria, and the sources and methods of selection of participants | Methods: Participants; Methods: Clusters; Methods: Data collection, questionnaire and outcomes |
| Variables | 7 | Clearly define all outcomes, exposures, predictors, potential confounders, and effect modifiers. Give diagnostic criteria, if applicable | Methods: Data collection, questionnaire and outcomes; Supplementary materials: Development of knowledge test score questions |
| Data sources/ measurement | 8* | For each variable of interest, give sources of data and details of methods of assessment (measurement). Describe comparability of assessment methods if there is more than one group | Methods: Data collection, questionnaire and outcomes; Supplementary materials: Development of knowledge test score questions |
| Bias | 9 | Describe any efforts to address potential sources of bias | Methods: Cluster and participant sampling |
| Study size | 10 | Explain how the study size was arrived at | Methods: Sample size |
| Quantitative variables | 11 | Explain how quantitative variables were handled in the analyses. If applicable, describe which groupings were chosen and why | Methods: Statistical analyses |
| Statistical methods | 12 | (*a*) Describe all statistical methods, including those used to control for confounding | Methods: Statistical analyses |
|  |  | (*b*) Describe any methods used to examine subgroups and interactions | Methods: Statistical analyses |
|  |  | (*c*) Explain how missing data were addressed | Methods: Statistical analyses |
|  |  | (*d*) If applicable, describe analytical methods taking account of sampling strategy | Methods: Statistical analyses |
|  |  | (*e*) Describe any sensitivity analyses | Methods: Statistical analyses |
| Results | | | |
| Participants | 13* | (a) Report numbers of individuals at each stage of study—eg numbers potentially eligible, examined for eligibility, confirmed eligible, included in the study, completing follow-up, and analysed | Results: Cluster size, number of participants and participants’ characteristics |
|  |  | (b) Give reasons for non-participation at each stage | NA |
|  |  | (c) Consider use of a flow diagram | NA |
| Descriptive data | 14* | (a) Give characteristics of study participants (eg demographic, clinical, social) and information on exposures and potential confounders | Results: Cluster size, number of participants and participants’ characteristics |
|  |  | (b) Indicate number of participants with missing data for each variable of interest | NA |
| Outcome data | 15* | Report numbers of outcome events or summary measures | Results: Levels of awareness of antibiotics and antibiotic resistance; Association between socio-demographic characteristics and reported awareness of antibiotics; Results: Association between socio-demographic characteristics and correct knowledge about antibiotics, antibiotic resistance, and the usage of antibiotics from a One Health perspective; Tables 2-4 |
| Main results | 16 | (*a*) Give unadjusted estimates and, if applicable, confounder-adjusted estimates and their precision (eg, 95% confidence interval). Make clear which confounders were adjusted for and why they were included | Results: Levels of awareness of antibiotics and antibiotic resistance; Association between socio-demographic characteristics and reported awareness of antibiotics; Results: Association between socio-demographic characteristics and correct knowledge about antibiotics, antibiotic resistance, and the usage of antibiotics from a One Health perspective; Tables 2-4 |
|  |  | (*b*) Report category boundaries when continuous variables were categorized | Results: Levels of awareness of antibiotics and antibiotic resistance; Association between socio-demographic characteristics and reported awareness of antibiotics; Results: Association between socio-demographic characteristics and correct knowledge about antibiotics, antibiotic resistance, and the usage of antibiotics from a One Health perspective; Tables 2-4 |
|  |  | (*c*) If relevant, consider translating estimates of relative risk into absolute risk for a meaningful time period | Absolute differences are presented for all outcomes |
| Other analyses | 17 | Report other analyses done—eg analyses of subgroups and interactions, and sensitivity analyses | Results: Levels of awareness of antibiotics and antibiotic resistance; Association between socio-demographic characteristics and reported awareness of antibiotics; Results: Association between socio-demographic characteristics and correct knowledge about antibiotics, antibiotic resistance, and the usage of antibiotics from a One Health perspective; Tables 2-4 |
| Discussion | | | |
| Key results | 18 | Summarise key results with reference to study objectives | Discussion: Reported awareness of antibiotics and antibiotic resistance; Results: Association between socio-demographic characteristics and awareness of antibiotics and correct knowledge about antibiotics, antibiotic resistance, and the usage of antibiotics from a One Health perspective |
| Limitations | 19 | Discuss limitations of the study, taking into account sources of potential bias or imprecision. Discuss both direction and magnitude of any potential bias | Discussion: Strengths and limitations |
| Interpretation | 20 | Give a cautious overall interpretation of results considering objectives, limitations, multiplicity of analyses, results from similar studies, and other relevant evidence | Discussion: Reported awareness of antibiotics and antibiotic resistance; Discussion: Association between socio-demographic characteristics and awareness of antibiotics and correct knowledge about antibiotics, antibiotic resistance, and the usage of antibiotics from a One Health perspective; Discussion: Conclusions |
| Generalisability | 21 | Discuss the generalisability (external validity) of the study results | Discussion: Reported awareness of antibiotics and antibiotic resistance; Discussion: Association between socio-demographic characteristics and awareness of antibiotics and correct knowledge about antibiotics, antibiotic resistance, and the usage of antibiotics from a One Health perspective; Discussion: Conclusions |
| Other information | | | |
| Funding | 22 | Give the source of funding and the role of the funders for the present study and, if applicable, for the original study on which the present article is based | Funding |
| See https://www.strobe-statement.org/ | | | |

# Descriptive estimands

Following the descriptive epidemiology framework ^1^ used in this study we define our descriptive estimands, i.e. the quantities that our inferential analyses target, here.

| **Target population** | **Outcome** | **Summary measure** | **Auxiliary variables** |
| --- | --- | --- | --- |
| Adult (≥18) rural community members in Cumilla district in 2022 | Binary response to the question “have you ever heard of a type of medicine known as an antibiotic or antibiotics?” – Yes (1) or No/Don’t know (0) | Percentage (frequency/total) | We will estimate the summary measure for the following subgroups within the target population and also estimate the difference in the outcome, on the percentage point scale, between the subgroups:   - Sex (Female/Male) - Age (18-25/26-32/33-40/41-55/56+) - Education level (No formal education or incomplete primary/Primary or incomplete secondary/Secondary or incomplete higher/Higher) - Worked in the past 30 days? (Yes/No) - Does the household own any animals? (Yes/No) |
| Adult (≥18) rural community members in Cumilla district in 2022 who responded “yes” to the question “have you ever heard of a type of medicine known as an antibiotic or antibiotics?” | Binary response to the question “have you heard of any of the terms ‘antibiotic resistance’, ‘antimicrobial resistance’ or ‘drug resistance?” – Yes (1) or No/Don’t know (0) | Percentage (frequency/total) | NA |
| Adult (≥18) rural community members in Cumilla district in 2022 who responded “yes” to the question “have you ever heard of a type of medicine known as an antibiotic or antibiotics?” | The sum of the number of correct or appropriate responses to 25 questions about correct knowledge about antibiotics and ABR and appropriate usage of antibiotics in relation to human health. | Percentage score (i.e. number of correct answers/total number of answers x 100) | We will estimate the summary measure for the following subgroups within the target population and also estimate the difference in the outcome, on the percentage point scale, between the subgroups:   - Sex (Female/Male) - Age (18-25/26-32/33-40/41-55/56+) - Education level (No formal education or incomplete primary/Primary or incomplete secondary/Secondary or incomplete higher/Higher) - Worked in the past 30 days? (Yes/No)   Does the household own any animals? (Yes/No) |
| Adult (≥18) rural community members in Cumilla district in 2022 who responded “yes” to the question “have you ever heard of a type of medicine known as an antibiotic or antibiotics?” | The sum of the number of correct or appropriate responses to 20 questions about correct knowledge about antibiotics and ABR and appropriate usage of antibiotics in relation to animal health and the environment. | Percentage score (i.e. number of correct answers/total number of answers x 100) | We will estimate the summary measure for the following subgroups within the target population and also estimate the difference in the outcome, on the percentage point scale, between the subgroups:   - Sex (Female/Male) - Age (18-25/26-32/33-40/41-55/56+) - Education level (No formal education or incomplete primary/Primary or incomplete secondary/Secondary or incomplete higher/Higher) - Worked in the past 30 days? (Yes/No)   Does the household own any animals? (Yes/No) |

# Methods: additional details

## Sample size

Due to logistical constraints around how many intervention clusters we believed we can implement in, we decided that we could have a maximum of 25 clusters per treatment group. As we plan to sample participants from two villages per cluster, and as we plan to sample an equal number of female and male participants per village, we also decided to sample an even number of participants per cluster. Following discussion among the trial team we decided that for both primary outcomes we would look at cluster-level treatment effects ^2^, which will mean aggregating the outcome to the cluster level by computing the mean of all survey participants’ percentage knowledge scores for each cluster (which we refer to as the cluster-level mean percentage knowledge score below). Based on discussion among the trial team we targeted being able to detect if the cluster-level mean percentage knowledge score for both outcomes was 15 percentage points or greater in the intervention group compared to the control group. We conservatively assumed an intracluster correlation coefficient of 0.3 for both primary outcomes, based on some related data from this setting in our previous survey study ^3^. We used the approach of Horzo to estimate plausible values for the overall variance for each primary outcome ^4^: 40 for the knowledge score measuring the level of correct knowledge about antibiotics and ABR and appropriate usage of antibiotics in relation to human health, and 25 for the knowledge score measuring the level of correct knowledge about antibiotics and ABR and appropriate usage of antibiotics in relation to animal health and the environment.

Based on these inputs and using the approach of ^5^ (based on formula 2 and assuming a cluster-level analysis), we then looked at a range of sample size scenarios. We adjusted the standard alpha level of 0.05 to 0.025, following a Bonferroni approach to account for the multiple testing of two primary outcomes. From these results we will aim to sample 44 participants per cluster (22 per village), so 1,100 participants across 25 clusters per treatment group and 2,200 participants across all 50 trial clusters, for the baseline and endline surveys. Conditional on these assumptions, when comparing between the treatment groups at endline we will have 90% power to detect a 14-percentage-point or greater difference between each group's mean cluster-level percentage knowledge score regarding correct knowledge about antibiotics and ABR and appropriate usage of antibiotics in relation to human health, and 90% power to detect a 11-percentage-point or greater difference between each group's mean cluster-level percentage knowledge score regarding correct knowledge about antibiotics and ABR and appropriate usage of antibiotics in relation to animal health and the environment.

## Setting

**Table S1. Population sizes and literacy levels for trial subdistricts**

| **Subdistrict** | **2011 population level** | **2011 literacy %** |
| --- | --- | --- |
| Homna | 169,728 | 39.7 |
| Daudkandi | 290,897 | 50.7 |
| Barura | 337,588 | 52 |
| Brahmanpara | 165,759 | 54.7 |
| Burichang | 250,708 | 57 |

## Clusters

As the baseline survey was part of a cluster randomised controlled trial our sampling was clustered. We defined our trial clusters as the population catchment areas of community clinics (CCs), which are government-run, basic, primary-healthcare facilities that serve rural communities across Bangladesh. The population catchment areas of CCs usually cover 4-6 (but sometimes >10) villages and around 6,000 individuals living in those villages. We chose the catchment areas of CCs for our clusters as the community dialogue approach involves running community dialogues - community-based and community-led interactive education sessions - within all the villages covered by the CC, and because the community dialogues are delivered by voluntary facilitators from the CC catchment communities, and these facilitators are supported by supervisors in the public health system, including the healthcare providers who work in the CCs. To maximise generalisability we did not have any cluster eligibility criteria, and so all clusters were eligible for participation in the trial.

## Cluster sampling

According to the available government data there were 144 CCs within our 5 chosen subdistricts. For the trial we aimed to stratify the cluster sampling by subdistrict with an equal number (10) of clusters sampled per subdistrict. However, to try and reduce the impact of any contamination between intervention and control clusters we also restricted the cluster sampling by maximising the linear distance between all clusters sampled within a subdistrict, such that there were still ≥10 clusters per subdistrict available for selection. Subject to this restriction we then randomly selected 10 clusters from the clusters available. In practice, in the 4 subdistricts other than Homna the minimum possible distance between selected CCs was 3 km, but in Homna, due to the CC density, this minimum distance had to be relaxed to 2.5 km to ensure ≥10 clusters were available.

## Participant sampling

For the baseline household survey we used a pragmatic, multi-stage sampling approach to select participants, following the method of the older WHO Expanded Program of Immunisation cluster sampling “spin the pen” approach. Within each subdistrict and cluster a team of six interviewers aimed to sample 22 participants from the village that the cluster's CC was located within, and a further 22 participants from the nearest adjacent village in the same cluster (or if there is just one village in the cluster then we aimed to sample all 44 participants from that village).

To sample participants from each selected village the data collection team located the approximate centre of the village and then spun a pen to select a random direction. They then walked a transect in that direction and sketch-mapped all households immediately either side of the transect line until they reached the edge of the village. They then numbered all the sketched households and, using a random number table, selected a random household from those listed. They then visited that household, located the first available eligible individual, informed them about the study and sought their consent to participate in the survey. If they agreed they were then interviewed. They then located the next nearest household, followed by the next nearest household in relation to that one, and so on, until they reached the fifth next nearest household from the one previously sampled. They then sampled and interviewed the first available eligible individual at this household who was of the opposite sex to the first participant. This sex switching was repeated for each subsequent participant to maintain a 1:1 female:male ratio.

They then repeated this skip selection process, avoiding households previously sampled, until they selected the required number of participants for that village. If an eligible and selected individual declined to participate they then tried to recruit another eligible individual in the household or moved onto the next nearest household. Where it was not possible to sample 22 or 44 individuals from separate households based on the household skip pattern, due to a lack of households, then modified the skip pattern accordingly.

## Questionnaire

Below we provide the questionnaire (in English, but this was translated into Bangla for the survey) that was used to collect the data used in this study, including the questions used to generate the four outcomes assessed in this study and the questions we asked about respondents’ socio-demographic characteristics.

### Socio-demographic characteristics questions

| Gender | Male |
| --- | --- |
|  | Female |
|  | Other *(please specify_____)* |
| How old are you? | \|  \|  \| \| --- \| --- \|   *(in completed years)* |
| How many family members currently live in your household? | \|  \|  \| \| --- \| --- \|   *(in numbers)* |
| What is your religion? | Muslim |
|  | Hindu |
|  | Christian |
|  | Buddhist |
|  | Others  (Please specify) |
| What is your last level of education you have had at an educational institution? | No formal education |
|  | Less than primary/equivalent |
|  | Primary completed/equivalent |
|  | Less than secondary/equivalent |
|  | Secondary completed/equivalent |
|  | Higher secondary/equivalent |
|  | Graduation/equivalent completed |
|  | Post-graduation and above |
| Have you done any work for cash or in-kind payment in the last 30-days? | Yes |
|  | No |
| What type of work was this, and if you have done more than one type of work, please just tell me the type you did most often? | Work on own farm or as a share cropper |
|  | Day/unskilled laborer (domestic, agricultural) |
|  | Skilled worker (long term contracted laborer) |
|  | Own business |
|  | Rickshaw/van puller |
|  | Driver (autorickshaw/CNG/Bus/Truck) |
|  | Government Service holder/professionals |
|  | Farm owner (agro, fisheries, chicken, duck, cow) |
|  | Tailor |
|  | Teacher |
|  | Tuition |
|  | Garment worker |
|  | Restaurant worker |
|  | Electrician |
|  | Mechanic |
|  | Travelling salesperson |
|  | Barber |
|  | Weaver |
|  | Immigrant |
|  | Home-maker |
|  | Retired Person |
|  | Shopkeeper |
|  | Handcraft-Seller |
|  | Private Service holder/professional |
|  | Others  *(Please specify_________)* |
| Do you or your family currently own any poultry, cattle, goats, sheep or fish for producing food, or to eat them, or to sell or trade? | Yes |
|  | No |
| Do you or your family currently own any poultry for producing food, or to eat them, or to sell or trade? | Yes |
|  | No |
| Do you or your family currently own any cattle for producing food, or to eat them, or to sell or trade? | Yes |
|  | No |
| Do you or your family currently own any goats/sheep for producing food, or to eat them, or to sell or trade? | Yes |
|  | No |
| Do you or your family currently own any fish to eat them, or to sell or trade? | Yes |
|  | No |

### Awareness of antibiotics outcome question

| Have you ever heard of a type of medicine known as an antibiotic or antibiotics? | Yes |
| --- | --- |
|  | No |
|  | Don’t know |

### Awareness of antibiotic resistance outcome question

See question 17 in “General and human-health-related knowledge questions on antibiotics and antibiotic resistance” below.

### Knowledge outcomes questions

#### General and human-health-related knowledge questions on antibiotics and antibiotic resistance

Responses considered correct/appropriate for the purpose of scoring the knowledge test questions are in **bold**.

| Can antibiotics treat any illness? | Yes |
| --- | --- |
|  | **No** |
|  | Don’t know |
| Can antibiotics treat illnesses caused by viruses? | Yes |
|  | **No** |
|  | Don’t know |
| Can antibiotics treat illnesses caused by bacteria? | **Yes** |
|  | No |
|  | Don’t know |
| Can antibiotics treat illnesses not caused by viruses or bacteria or other microbes, like diabetes or high blood pressure (also called hypertension)? | Yes |
|  | **No** |
|  | Don’t know |
| Is there just one type of antibiotic? | Yes |
|  | **No** |
|  | Don’t know |
| Can antibiotics be given as a tablet? | **Yes** |
|  | No |
|  | Don’t know |
| Can antibiotics be given as an injection? | **Yes** |
|  | No |
|  | Don’t know |
| Can antibiotics be given as a cream/ointment? | **Yes** |
|  | No |
|  | Don’t know |
| Are more expensive antibiotics always better at treating illness than less expensive antibiotics? | Yes |
|  | **No** |
|  | Don’t know |
| Assume that you became ill and when you went to get treatment from a drug seller, they recommended that you buy some antibiotics from them. Would you trust their advice and buy the antibiotics recommended or not trust their advice and not buy the antibiotics from them? | Trust their advice |
|  | **Not trust their advice** |
|  | Don’t know |
| Assume that you became ill and thought you needed antibiotics to get better and so you went to a qualified healthcare provider for treatment, such as a CHCP at your local community clinic, or a doctor at Upazila Health Complex or a qualified doctor at a private clinic. If they told you that you did not need antibiotics, would you trust their advice or ignore their advice and seek antibiotics elsewhere? | **Trust their advice** |
|  | Not trust their advice |
|  | Don’t know |
| Is it appropriate or inappropriate to buy antibiotics to keep at home in case you or someone else in the household gets ill in the future? | Appropriate |
|  | **Inappropriate** |
|  | Don’t know |
| If you or someone in your household gets ill and is treated with antibiotics and some are left over, is it appropriate or inappropriate to keep those antibiotics at home in case you or someone else in the household gets ill in the future? | Appropriate |
|  | **Inappropriate** |
|  | Don’t know |
| Assume that you are taking antibiotics for an illness and you start to feel better after taking just half the course you were advised to take. Is it appropriate or inappropriate to not take any of the rest of the course? | Appropriate |
|  | **Inappropriate** |
|  | Don’t know |
| Assume that you are ill and have been prescribed a course of antibiotics by a qualified healthcare provider, such as a community health care provider (CHCP) or a doctor at the Upazilla Health Complex or a qualified doctor at a private clinic, which you have been taking. If the antibiotics don’t seem to be helping do you think it is appropriate or inappropriate to take more antibiotics each day than advised without speaking to the provider first? | Appropriate |
|  | **Inappropriate** |
|  | Don’t know |
| Assume that you are ill and have been prescribed a course of antibiotics by a qualified healthcare provider, such as a community health care provider (CHCP) or doctor at the Upazilla Health Complex or a qualified doctor at a private clinic. Is it appropriate or inappropriate to keep the prescription for use again in the future if you had the same symptoms? | Appropriate |
|  | **Inappropriate** |
|  | Don’t know |
| Have you heard of any of the terms *“antibiotic resistance”*, *“antimicrobial resistance”* or *“drug resistance”*? | Yes |
|  | No |
|  | Don’t know |
| Does antibiotic resistance happen when your body becomes resistant to an antibiotic? | Yes |
|  | **No** |
|  | Don’t know |
| Does antibiotic resistance happen when the microbes causing an infection become resistant to the antibiotic which can no longer kill them? | **Yes** |
|  | No |
|  | Don’t know |
| Can people who are not taking antibiotics get antibiotic resistant diseases, such as drug-resistant TB? | **Yes** |
|  | No |
|  | Don’t know |
| In Bangladesh, are antibiotic resistant diseases about as common now as in the past, or are they becoming less common or are they becoming more common? | Same |
|  | Less common |
|  | **More common** |
|  | Don’t know |
| Which one do you think is easier to treat with antibiotics? An antibiotic resistant infection. An infection that is treatable with an antibiotic. Neither one is more treatable than the other. | An antibiotic resistant infection |
|  | **An infection that is treatable with an antibiotic** |
|  | Neither one is more treatable than the other. |
|  | Don’t know |
| Can antibiotic resistant diseases spread from person to person? | **Yes** |
|  | No |
|  | Don’t know |
| Can vaccinations help prevent the diseases from occurring? | **Yes** |
|  | No |
|  | Don’t know |
| Can antibiotics save lives when used properly? | **Yes** |
|  | No |
|  | Don’t know |

#### Animal-health-related knowledge questions on antibiotics and antibiotic resistance

| Can antibiotics treat all illnesses of animals? | Yes |
| --- | --- |
|  | **No** |
|  | Don’t know |
| Can some diseases be spread between people and animals and between animals and people? | **Yes** |
|  | No |
|  | Don’t know |
| Can people get diseases that have become resistant to treatment by antibiotics from animals? | **Yes** |
|  | No |
|  | Don’t know |
| Assume you owned a cow that became ill and when you asked the local village doctor for help, they told you to give the cow some antibiotics. Would it be appropriate or inappropriate to follow their advice? | Appropriate |
|  | **Inappropriate** |
|  | Don’t know |
| Assume you owned a cow that became ill and you thought it needed antibiotics to get better, but when you went to the nearest upazila veterinary hospital you were told that antibiotics would not help. Would you trust their advice or seek antibiotics elsewhere? | **Trust their advice** |
|  | Not trust their advice |
|  | Don’t know |
| Assume you owned a cow. Is it appropriate or inappropriate to buy antibiotics to keep inside the home in case the cow gets ill in the future? | Appropriate |
|  | **Inappropriate** |
|  | Don’t know |
| Assume you owned a cow. If the cow became ill and needed to be treated with antibiotics and some are left over, is it appropriate or inappropriate to keep those antibiotics inside home in case the cow gets ill in the future? | Appropriate |
|  | **Inappropriate** |
|  | Don’t know |
| Assume you were taking antibiotics for an illness but you also owned a cow that became ill with similar symptoms. Would it be appropriate or inappropriate to give some of your antibiotics to your animal? | Appropriate |
|  | **Inappropriate** |
|  | Don’t know |
| Assume you owned a cow that became ill and you were treating it with antibiotics, and you also became ill with similar symptoms. Would it be appropriate or inappropriate to take some of your cow’s antibiotics for your own illness? | Appropriate |
|  | **Inappropriate** |
|  | Don’t know |
| Assume you owned a cow that became ill and you were advised to treat it with antibiotics by a qualified vet, such as a Upazilla livestock officer or a livestock extension officer from a Upazila veterinary hospital, and the animal seemed to recover after you gave it just half the course you were advised to give it. Would it be appropriate or inappropriate to stop treating it and not give it the rest of the course? | Appropriate |
|  | **Inappropriate** |
|  | Don’t know |
| Assume you owned a cow that became ill and you were advised to treat it with antibiotics by a qualified vet, such as a Upazilla livestock officer or a livestock extension officer from a Upazila veterinary hospital. If the antibiotics didn’t seem to be helping, would it be appropriate or inappropriate to give the cow more of the antibiotics each day than you were advised to without speaking to the vet again first? | Appropriate |
|  | **Inappropriate** |
|  | Don’t know |
| Can giving healthy animals antibiotics, for example in their feed, to make them grow better or produce more milk or eggs, cause antibiotic resistant infections to happen in the animals? | **Yes** |
|  | No |
|  | Don’t know |
| Can giving healthy animals antibiotics, for example in their feed or water, to try and stop them from getting any diseases they don’t currently have in the future, cause antibiotic resistant infections to happen in the animals, which can be hard to treat with antibiotics? | **Yes** |
|  | No |
|  | Don’t know |
| If an animal is given an antibiotic and someone eats the animals’ meat, eggs or milk within a few days of the animal being given the antibiotic can the antibiotic be passed into their body by eating the animal’s meat, eggs or milk? | **Yes** |
|  | No |
|  | Don’t know |
| If an animal is given an antibiotic does the antibiotic also get spread into the environment (for example into ponds or the soil) in the animal’s faeces and urine? | **Yes** |
|  | No |
|  | Don’t know |
| Can you help to stop the spread of antibiotic resistant infections by keeping animals away from open water sources, like ponds? | **Yes** |
|  | No |
|  | Don’t know |
| Can you help to stop the spread of antibiotic resistant infections by not giving healthy animals feed containing antibiotics? | **Yes** |
|  | No |
|  | Don’t know |
| Can vaccinating animals stop them from getting certain infectious diseases? | **Yes** |
|  | No |
|  | Don’t know |
| Can vaccinating animals stop them from spreading diseases, including antibiotic resistant diseases, to humans? | **Yes** |
|  | No |
|  | Don’t know |
|  |  |
|  |  |
| Can antibiotics ever save animals’ lives if used properly according to the veterinary doctors’ advice? | **Yes** |
|  | No |
|  | Don’t know |
|  |  |

# Statistical analyses: additional details

## Main analyses

In Table S2 below we describe in full the approach taken to compute all inferential results for each outcome analysed in relation to the broad research questions, which we reiterate here. These relate to the target population of adult (≥18) rural community members in Cumilla district in 2022. First, what percentage of individuals report being aware of the existence antibiotics as a type of medicine, and what associations exist between key socio-demographic characteristics and antibiotic awareness levels. Second, among those who report awareness of antibiotics what percentage report being aware of the existence of ABR? Third, among those who report awareness of antibiotics what is the level of correct knowledge about antibiotics, antibiotic resistance, and the usage of antibiotics from a One Health perspective (in relation to human health, animal health and the environment), and what associations exist between key socio-demographic characteristics and knowledge levels? The sample sizes for all the analyses can be found in the relevant tables in the main paper (Tables 2-5).

**Table S2. Outcome and estimand specific analysis details**

| **Outcome** | **Estimand** | **Method** |
| --- | --- | --- |
| **Estimands targeting outcome means in the entire target populations** | | |
| Self-reported awareness of antibiotics (binary: yes/no or don’t know) | The mean percentage of individuals reporting awareness of antibiotics among adults (≥18) in rural communities in Cumilla district in 2022. | Fit a GLM with a Bernoulli distribution and a logit link to the outcome with one covariate for subdistrict^[[1]](#footnote-1)^ (the sampling strata), using the *glm()^[[2]](#footnote-2)^* function. Use the *avg_predictions()^[[3]](#footnote-3)^* function to generate outcome predictions for all observations on the response (probability) scale and compute their average, along with the associated 95% confidence intervals. Transform the average predicted probability of the outcome and associated 95% confidence intervals to the percentage scale by multiplying by 100. |
| Self-reported awareness of ABR (binary: yes/no or don’t know) | The mean percentage of individuals reporting awareness of ABR among adults (≥18) in rural communities in Cumilla district in 2022. | Fit a GLM with a Bernoulli distribution and a logit link to the outcome with one covariate for subdistrict^1^ (the sampling strata), using the *glm()^2^* function. Use the *avg_predictions()*^3^ function to generate outcome predictions for all observations on the response (probability) scale and compute their average, along with the associated 95% confidence intervals. Transform the average predicted probability of the outcome and associated 95% confidence intervals to the percentage scale by multiplying by 100. |
| **Estimands targeting outcome means in subgroups within the target populations** | | |
| Self-reported awareness of antibiotics (binary: yes/no or don’t know) | The mean percentage of individuals within each socio-demographic subgroup (as defined by the categories of each socio-demographic variable in the analyses) reporting awareness of antibiotics among adults (≥18) in rural communities in Cumilla district in 2022. | Fit a GLM with a Bernoulli distribution and a logit link to the outcome with one covariate measuring one of the socio-demographic characteristics of interest plus a covariate for subdistrict^1^ (the sampling strata), using the *glm()*^2^ function. Use the *avg_predictions()*^3^ function to generate outcome predictions for all observations on the response (probability) scale and compute their average for each of the subgroups as defined by the categories of the socio-demographic covariate, along with all associated 95% confidence intervals. Transform the subgroup-specific averages of the predicted probabilities of the outcome and associated 95% confidence intervals from the probability scale to the percentage scale. Repeat this process for every socio-demographic characteristic of interest. |
| Correct knowledge about antibiotics and ABR and appropriate usage of antibiotics in relation to human health (the number of correct answers to 25 binary/multiple choice questions) | The mean percentage test score within each socio-demographic subgroup (as defined by the categories of each socio-demographic variable in the analyses) among adults (≥18) in rural communities in Cumilla district in 2022, who also reported awareness of antibiotics. | Fit a GLM with a binomial distribution and a logit link to the outcome (n correct responses out of N trials [the total number of questions]) with one covariate measuring one of the socio-demographic characteristics of interest plus a covariate for subdistrict^1^ (the sampling strata), using the *glm()*^2^ function. Use the *avg_predictions()*^3^ function to generate outcome predictions for all observations on the response (probability) scale and compute their average for each of the subgroups as defined by the categories of the socio-demographic covariate, along with all associated 95% confidence intervals. Transform the subgroup-specific averages of the predicted probabilities of the outcomes and associated 95% confidence intervals to the percentage scale. Repeat this process for every socio-demographic characteristic of interest. |
| Correct knowledge about antibiotics and ABR and appropriate usage of antibiotics in relation to animal health and the environment (number of correct answers to 20 binary/multiple choice questions) | The mean percentage test score within each socio-demographic subgroup (as defined by the categories of each socio-demographic variable in the analyses) among adults (≥18) in rural communities in Cumilla district in 2022, who also reported awareness of antibiotics. | Fit a GLM with a binomial distribution and a logit link to the outcome (n correct responses out of N trials [the total number of questions]) with one covariate measuring one of the socio-demographic characteristics of interest plus a covariate for subdistrict^1^ (the sampling strata), using the *glm()*^2^ function. Use the *avg_predictions()*^3^ function to generate outcome predictions for all observations on the response (probability) scale and compute their average for each of the subgroups as defined by the categories of the socio-demographic covariate, along with all associated 95% confidence intervals. Transform the subgroup-specific averages of the predicted probabilities of the outcomes and associated 95% confidence intervals to the percentage scale. Repeat this process for every socio-demographic characteristic of interest. |
| **Estimands targeting differences in outcome means between subgroups within the target populations** | | |
| Self-reported awareness of antibiotics (binary: yes/no or don’t know) | The difference (on the additive scale) in the mean percentage of individuals reporting awareness of antibiotics within each socio-demographic subgroup of interest compared to the reference subgroup (with subgroups defined by the categories of each socio-demographic variable in the analyses – see Table 2 for the reference subgroups chosen for each socio-demographic variable) among adults (≥18) in rural communities in Cumilla district in 2022.  T2-4 | Fit a GLM with a Bernoulli distribution and a logit link to the outcome with one covariate measuring one of the socio-demographic characteristics of interest plus a covariate for subdistrict^1^ (the sampling strata), using the *glm()*^2^ function. Use the *avg_comparisons()*^3^ function to generate outcome predictions for all observations on the response (probability) scale, compute their average for each of the subgroups as defined by the categories of the socio-demographic covariate, and finally compute the differences, on the additive scale, between each subgroup’s average predicted outcome compared to the average predicted outcome for the designated reference subgroup, along with all associated 95% confidence intervals for those differences. Transform the differences between the relevant subgroup-specific average predicted probabilities of the outcome and associated 95% confidence intervals to the percentage point scale by multiplying by 100. Repeat this process for every socio-demographic characteristic of interest. |
| Correct knowledge about antibiotics and ABR and appropriate usage of antibiotics in relation to human health (the number of correct answers to 25 binary/multiple choice questions) | The difference (on the additive scale) in the mean percentage of individuals reporting awareness of antibiotics within each socio-demographic subgroup of interest compared to the reference subgroup (with subgroups defined by the categories of each socio-demographic variable in the analyses – see Table 3 for the reference subgroups chosen for each socio-demographic variable) among adults (≥18) in rural communities in Cumilla district in 2022. | Fit a GLM with a binomial distribution and a logit link to the outcome (n correct responses out of N trials [the total number of questions]) with one covariate measuring one of the socio-demographic characteristics of interest plus a covariate for subdistrict^1^ (the sampling strata), using the *glm()*^2^ function. Use the *avg_comparisons()*^3^ function to generate outcome predictions for all observations on the response (probability) scale, compute their average for each of the subgroups as defined by the categories of the socio-demographic covariate, and finally compute the differences, on the additive scale, between each subgroup’s average predicted outcome compared to the average predicted outcome for the designated reference subgroup, along with all associated 95% confidence intervals for those differences. Transform the differences between the relevant subgroup-specific average predicted probabilities of the outcome and associated 95% confidence intervals to the percentage point scale. Repeat this process for every socio-demographic characteristic of interest. |
| Correct knowledge about antibiotics and ABR and appropriate usage of antibiotics in relation to animal health and the environment (number of correct answers to 20 binary/multiple choice questions) | The difference (on the additive scale) in the mean percentage of individuals reporting awareness of antibiotics within each socio-demographic subgroup of interest compared to the reference subgroup (with subgroups defined by the categories of each socio-demographic variable in the analyses – see Table 4 for the reference subgroups chosen for each socio-demographic variable) among adults (≥18) in rural communities in Cumilla district in 2022. | Fit a GLM with a binomial distribution and a logit link to the outcome (n correct responses out of N trials [the total number of questions]) with one covariate measuring one of the socio-demographic characteristics of interest plus a covariate for subdistrict^1^ (the sampling strata), using the *glm()*^2^ function. Use the *avg_comparisons()*^3^ function to generate outcome predictions for all observations on the response (probability) scale, compute their average for each of the subgroups as defined by the categories of the socio-demographic covariate, and finally compute the differences, on the additive scale, between each subgroup’s average predicted outcome compared to the average predicted outcome for the designated reference subgroup, along with all associated 95% confidence intervals for those differences. Transform the differences between the relevant subgroup-specific average predicted probabilities of the outcome and associated 95% confidence intervals to the percentage point scale. Repeat this process for every socio-demographic characteristic of interest. |
| ABR = antibiotic resistance.  All confidence intervals based on “HC3” cluster robust standard errors^[[4]](#footnote-4)^. | | |

# Additional comparisons between age and education level subgroups

**Table S3. Additional comparisons of differences in the percentage of self-reported awareness of antibiotics between age and education level subgroups**

| **Comparison** | **Percentage point difference compared to reference subgroup (95% CI)^a^** |
| --- | --- |
| Age |  |
| (56+) - (41 to 55) | -3.6 (-10.6, 3.4) |
| (56+) - (33 to 40) | -10.4 (-17.5, -3.4) |
| (56+) - (26 to 32) | -13.8 (-20.3, -7.4) |
| Education level |  |
| (Higher) - (Secondary/incomplete higher) | 3 (-0.1, 6.1) |
| (Higher) - (Primary/incomplete secondary) | 11.3 (8.2, 14.4) |
| n = 2160 for all models/results presented. This was all individuals who agreed to participate in the survey out of the 2187 who were approached (response rate = 98.8%).  Outcome variable = a response of “Yes” (1) or “No/don’t know” (0) to the question “Have you ever heard of a type of medicine known as an antibiotic or antibiotics?”  ^a^ Each difference reflects the difference (on the additive scale) between the mean (model-predicted) percentage of individuals reporting awareness of antibiotics for the subgroups indicated compared to the reference subgroups indicated (with units of percentage points). All confidence intervals are based on “HC3” cluster robust standard errors. See Table S2 footnote 4 for justification. | |

**Table S4. Additional comparisons of differences in the mean percentage test score for correct knowledge about antibiotics and ABR and appropriate usage of antibiotics in relation to human health between age and education level subgroups (among individuals reporting awareness of antibiotics)**

| **Comparison** | **Percentage point difference compared to reference subgroup (95% CI)^a^** |
| --- | --- |
| Age |  |
| (56+) - (41 to 55) | -0.7 (-2.7, 1.3) |
| (56+) - (33 to 40) | -0.2 (-2.2, 1.8) |
| (56+) - (26 to 32) | -0.8 (-3.1, 1.5) |
| Education level |  |
| (Higher) - (Secondary/incomplete higher) | 5.4 (3.6, 7.3) |
| (Higher) - (Primary/incomplete secondary) | 8.9 (7.1, 10.7) |
| n = 1774 for all models/results presented. This was all individuals who agreed to participate in the survey and reported being aware of antibiotics (82.1% of the 2160 individuals who were approached and agreed to participate in the survey).  Test comprised of 25 multiple-choice questions assessing individuals’ correct knowledge about antibiotics and ABR and appropriate usage of antibiotics in relation to human health. Each question response was assessed as either correct/appropriate or incorrect/inappropriate, with the overall test score (i.e. outcome variable values) being a simple sum of the number of correct/appropriate responses.  ^a^ Each difference reflects the difference (on the additive scale) between the mean (model-predicted) percentage test score for the subgroups indicated compared to the reference subgroups indicated (with units of percentage points). All confidence intervals are based on “HC3” cluster robust standard errors. See Table S2 footnote 4 for justification. | |

**Table S5. Additional comparisons of differences in the mean percentage test score for correct knowledge about antibiotics and ABR and appropriate usage of antibiotics in relation to animal health and the environment between age and education level subgroups (among individuals reporting awareness of antibiotics)**

| **Comparison** | **Percentage point difference compared to reference subgroup (95% CI)^a^** |
| --- | --- |
| Age |  |
| (56+) - (41 to 55) | -2.5 (-5.3, 0.2) |
| (56+) - (33 to 40) | -1.1 (-4.2, 2) |
| (56+) - (26 to 32) | -0.8 (-3.9, 2.2) |
| Education level |  |
| (Higher) - (Secondary/incomplete higher) | 2.2 (-0.2, 4.5) |
| (Higher) - (Primary/incomplete secondary) | 5.1 (3.1, 7.1) |
| n = 1774 for all models/results presented. This was all individuals who agreed to participate in the survey and reported being aware of antibiotics (82.1% of the 2160 individuals who were approached and agreed to participate in the survey).  Test comprised of 20 multiple-choice questions assessing individuals’ correct knowledge about antibiotics and ABR and appropriate usage of antibiotics in relation to animal health and the environment. Each question response was assessed as either correct/appropriate or incorrect/inappropriate, with the overall test score (i.e. outcome variable values) being a simple sum of the number of correct/appropriate responses.  ^a^ Each difference reflects the difference (on the additive scale) between the mean (model-predicted) percentage test score for the subgroups indicated compared to the reference subgroups indicated (with units of percentage points). All confidence intervals are based on “HC3” cluster robust standard errors. See Table S2 footnote 4 for justification. | |

# Author reflexivity statement

“Structured reflexivity statement to be completed with manuscript submissions from international research partnerships involving researchers from high- and low-to-middle-income countries. This describes 15 questions that should be addressed by corresponding authors on behalf of an international research partnership. The questions are intentionally open-ended and designed to address specific components of equitable research partnership. It may be that not all questions can be addressed (e.g. a small project with minimal or no funding) but researchers should be able to describe individual components that they have considered when developing their partnership” (10).

Study conceptualisation

1. How does this study address local research and policy priorities?

AMR has been identified as a major public health threat in Bangladesh. There is limited evidence about the knowledge and behaviour of people from rural communities in relation to antibiotics, antibiotic resistance, and the use of antibiotics from a One Health perspective. This study is part of a long-term project to develop and evaluate a community engagement intervention to tackle antimicrobial resistance in the context of rural Bangladeshi communities and has involved national and regional policy makers and stakeholders throughout. This specific study will provide evidence to help guide future tailoring of the community engagement intervention.

1. How were local researchers involved in study design?

All members of the in-country research team (RH, MBS, FF, KI, AR, MNI) were involved in all aspects of the study design, including the conceptualisation, and particularly in the data collection and data management aspects, for the wider COSTAR project that this study is a part of.

Research management

1. How has funding been used to support the local research team(s)?

The wider COSTAR project that this study is a part of was funded by the UK’s UKRI/GCRF collective fund. The in-country partner organisation ARK Foundation is the implementing partner for the intervention and carried out all data collection. They have therefore received sufficient funding given their role.

Data acquisition and analysis

1. How are research staff who conducted data collection acknowledged?

The in-country research staff hired to conduct data collection are acknowledged in the *Acknowledgements* section of the study protocol and this paper.

1. How have members of the research partnership been provided with access to study data?

All members of the research partnership had access to the study data, but it is managed by the in-country research team ARK Foundation.

1. How were data used to develop analytical skills within the partnership?

The in-country research team (RH, MBS, FF, KI, AR, MNI) led the development and management of the digital data collection tools, processes and development and management of the database. They also led the initial data cleaning of the database. Although no members of the in-country research team were involved in the analysis of the data used in this study they will be involved in the analysis of the data from the trial and wider project (both quantitative and qualitative).

Data interpretation

1. How have research partners collaborated in interpreting study data?

All members of the in-country research team (RH, MBS, FF, KI, AR, MNI) contributed to the interpretation of the results as presented in this paper.

Drafting and revising for intellectual content

1. How were research partners supported to develop writing skills?

All members of the in-country research team (RH, MBS, FF, KI, AR, MNI) contributed to the editing and development of this paper. More widely, members of the in-country research team (RH, MBS, FF, KI, AR, MNI) are also involved (as both first- and co-authors) on multiple other papers from the wider COSTAR project that this study is a part of.

1. How will research products be shared to address local needs?

The results of this study and the wider project will be shared by the in-country research team with the stakeholders involved in the development of the study, via research/policy briefs and stakeholder meetings.

Authorship

1. How is the leadership, contribution and ownership of this work by LMIC researchers recognised within the authorship?

All members of the in-country research team (RH, MBS, FF, KI, AR, MNI) involved in the elements of the wider COSTAR project that have been used in this study are included as co-authors on this paper. Although the first author for this paper is a member of the research partnership from the UK, members of the in-country research team are also involved (as both first- and co-authors) on multiple other papers from the wider COSTAR project that this study is a part of.

1. How have early career researchers across the partnership been included within the authorship team?

Within the in-country research team two early career researchers, MBS and FF, were involved in the development of the wider COSTAR project and were responsible for the day-to-day management and running of the trial. They are both co-authors on this paper.

1. How has gender balance been addressed within the authorship?

6/11 of the authors are female and 3/6 authors who are members of the in-country research team are female.

Training

1. How has the project contributed to training of LMIC researchers?

This data analysis and most of the writing for this paper was done by a researcher from the UK. Also, most the in-country research team involved in this paper are mid-level or senior-level researchers. However, the early career researchers (MBS and FF and others who were not involved in this paper) have had and will have the opportunity to increase their skills and knowledge around project management, data analysis and paper writing through their work on the wider COSTAR project.

Infrastructure

1. How has the project contributed to improvements in local infrastructure?

There are no direct impacts of this specific study on improvements in local infrastructure, but the wider COSTAR project that this study is a part of aims to evaluate whether a community engagement approach can improve community knowledge and practices around antimicrobial resistance. If this evidence is positive the hope is that this will facilitate the scale-up of the intervention, which would implement structures and processes to tackle antimicrobial resistance across rural Bangladesh.

Governance

1. What safeguarding procedures were used to protect local study participants and researchers?

For the wider project that this study was part of we developed a safeguarding plan, trained all members of the research team on safeguarding issues and our safeguarding processes, and created an in-country safeguarding lead role within the in-country research team.

# References

1. Lesko CR, Fox MP, Edwards JK. A Framework for Descriptive Epidemiology. *American Journal of Epidemiology* 2022;191(12):2063-70. doi: 10.1093/aje/kwac115

2. Kahan BC, Li F, Copas AJ, Harhay MO. Estimands in cluster-randomized trials: choosing analyses that answer the right question. *Int J Epidemiol* 2023;52(1):107-18. doi: 10.1093/ije/dyac131

3. Hicks JP, Latham SM, Huque R, et al. Antibiotic practices among household members and their domestic animals within rural communities in Cumilla district, Bangladesh: a cross-sectional survey. *Bmc Public Health* 2021;21(1) doi: 10.1186/s12889-021-10457-w

4. Hozo SP, Djulbegovic B, Hozo I. Estimating the mean and variance from the median, range, and the size of a sample. *BMC Medical Research Methodology* 2005;5(1):13. doi: 10.1186/1471-2288-5-13

5. Donner A, Birkett N, Buck C. Randomization by cluster. Sample size requirements and analysis. *Am J Epidemiol* 1981;114(6):906-14. doi: 10.1093/oxfordjournals.aje.a113261

1. To increase the precision of the estimates. [↑](#footnote-ref-1)
2. Base R function (R version 4.3.2): R Core Team (2023). R: A Language and Environment for Statistical Computing. R Foundation for Statistical Computing, Vienna, Austria. <https://www.R-project.org/>. [↑](#footnote-ref-2)
3. From marginaleffects package: Arel-Bundock V (2023). marginaleffects: Predictions, Comparisons, Slopes, Marginal Means, and Hypothesis Tests. R package version 0.16.0, <https://CRAN.R-project.org/package=marginaleffects>. [↑](#footnote-ref-3)
4. There is limited evidence but some evidence suggests that the HC3 adjustment is the best performing out of the most commonly available small sample adjustments (or none) for cluster robust standard errors, at least when fitting a Bernoulli GLM (with its canonical link) with one continuous covariate to small datasets, and specifically in terms of the confidence interval coverage rate for that covariate. See Zeileis A, Köll S, Graham N (2020). “Various Versatile Variances: An Object-Oriented Implementation of Clustered Covariances in R.” Journal of Statistical Software, 95(1), 1-36. doi:10.18637/jss.v095.i01. [↑](#footnote-ref-4)
