# Supplementary material for: Associations between sociodemographic characteristics and knowledge about antibiotics and antibiotic resistance and usage of antibiotics from a One Health perspective in rural Bangladesh: a descriptive cross-sectional study
Source: BMJ Open. 2025 Dec 18;15(12):e104131. doi: 10.1136/bmjopen-2025-104131 (PMC12716581; doi:10.1136/bmjopen-2025-104131)
Supplement: Supplementary file 2 [file bmjopen-2025-104131.reviewer_comments.pdf]

# PEER REVIEW HISTORY

BMJ Open publishes all reviews undertaken for accepted manuscripts. Reviewers are asked to complete a checklist review form and are provided with free text boxes to elaborate on their assessment. These free text comments are reproduced below.

## ARTICLE DETAILS

### Title (Provisional)

Associations between socio-demographic characteristics and knowledge about antibiotics and antibiotic resistance and usage of antibiotics from a One Health perspective in rural Bangladesh: a descriptive cross-sectional study

### Authors

Hicks, Joseph; Huque, Rumana; Fieroze, Fariza; Saify, Md Badruddin; Ensor, Tim; Islam, Khaleda; Latham, Sophia; Mitchell, Jessica; Rahman, Aninda; Islam, Md Nazmul; King, Rebecca

---

## VERSION 1 - REVIEW

---

| | |
|---|---|
| **Reviewer** | **1** |
| **Name** | **Hashmi, Furqan** |
| **Affiliation** | **University of the Punjab, Pharmacy Practice** |
| **Date** | **06-Jul-2025** |
| **COI** | **None** |

I have gone through the manuscript with interest that covers an important issue of AMR and antibiotic use, specially in the context of a LMIC.

---

| | |
|---|---|
| **Reviewer** | **2** |
| **Name** | **Golding , Sarah** |
| **Affiliation** | **University of Surrey, School of Psychology** |
| **Date** | **19-Jul-2025** |
| **COI** | **None** |

Manuscript Review - Associations between socio-demographic characteristics and knowledge about antibiotics, antibiotic resistance, and the use of antibiotics from a One Health perspective in rural Bangladesh: a descriptive cross-sectional study.

Overall comments

This manuscript reports a cross-sectional survey that examines – amongst the general adult population in rural communities in Bangladesh - their 1) awareness of antibiotics, 2) awareness of antibiotic resistance (ABR), 3) knowledge about use of antibiotics in humans and 4) knowledge about use of antibiotics in animals and in relation to the environment. The study has the potential to contribute to the literature – especially as it examines these issues from a One Health perspective - if some areas of the manuscript are sufficiently revised.

Overall, there is coherence to the manuscript – it is grounded in relevant literature, clearly written and easy for the reader to follow in most places, with a logical structure throughout. The manuscript offers originality by reporting on knowledge about appropriate use of antibiotics from a One Health perspective, within the context of rural communities in a lower-resource setting. The aims are clearly defined, with an appropriate study design and methods, and findings could have potential relevance for public health workers and researchers.

However, in its current draft, my recommendation is for major revisions, as I have some questions about how the statistical analyses are currently described and results presented. Within the Methods, there needs to be more clarity about the inferential analyses performed. Within the Results (or supplementary material), there needs to be additional descriptive and inferential statistics reported. I therefore cannot comment on the interpretations and conclusion at this stage. I have provided detailed comments below, which I think need to be addressed before the manuscript can be re-considered for publication.

Abstract

Concisely written – clear overview of study methods and key results

Introduction

Clear and well-written – succinctly sets out the contextual threat from ABR, the context for this specific study, and the study aims/research questions. The authors are transparent about how these data have been collected, why they are being analysed for this study, and how data will be used in the future.

Please can the authors address the following, to help strengthen the rationale for this study:

• Provide a brief explanation about why there is a need to assess awareness and knowledge levels by socio-demographic variables (i.e., a sentence or two of theoretical justification, grounded in evidence from other studies in similar contexts, about why there is an expectation that knowledge might vary by age, education etc.)

Methods

For the most part, the methods have been reported sufficiently, and in line with two checklists appropriate for the study design. There is a mostly sufficient overview provided in the main text, with extensive additional detail provided in supplementary materials.

I am not an expert on cluster sampling methodology, so cannot comment on potential limitations of the approach adopted in this study – but I can follow from the authors' reporting the steps taken to create their sample, so I am satisfied this has been reported with sufficient transparency, supported by a reasoned argument.

However, I did find the description and rationale for the inferential analyses confusing in places - there seems to be some incoherence between the Methods and Results, regarding analyses described in the Methods, but results not reported (or only partially reported). I am also not clear exactly what inferential analyses have been performed, and in places I am not sure if the authors have reported actual (observed) data or the expected (predicted) data calculated from models.

Please can the authors address / reflect on the following:

• Lines 245-252: There is reference made to two types of analysis – one looking at outcomes (e.g., awareness, knowledge) for the overall target population (i.e. the whole dataset?) and one looking at outcomes by sub-group (e.g., age, education etc) – yet, I cannot see reported in the main text or the supplemental material the descriptive statistics for the whole dataset (e.g., Table 2 reports awareness of antibiotics for the sub-groups only, and I cannot see values for the outcome for the sample as a whole?).

o Please add these whole sample data for all outcomes.

• Lines 245-252: The text indicates that all models built included subdistrict as an independent variable – but I cannot see anywhere in the main text or supplemental materials any outputs that include values for the subdistrict variable.

o Please report the relevant descriptive and inferential statistics for associations between subdistrict and the 4 outcomes.

• Lines 252-262: I find this section of text confusing and hard to follow. It is not clear exactly what analyses and what data have been reported in the Results. For this type of study, I would expect to see descriptives reported from the observed data, (i.e., we asked 2160 people a question and X% answered correctly) for the whole sample, and for the sub-groups – and then a separate set of inferential analyses (e.g., logistic regressions) to examine sub-group differences. Yet, as currently drafted (and in combination with the Table 2-4 footnotes, and the additional information in the supplements), this section of text in the Methods suggests to me that the authors have not reported e.g., the % of e.g., Females/Males reporting awareness from the actual data (i.e., descriptive stats – percentages / means - based on observed data) but have instead reported predicted percentages/means (from a predictive model? To estimate percentages/means in the target population, extrapolated from the sample population?). I also cannot locate the results of inferential tests of

difference in the results, such as p-values and effect sizes. I am not familiar with using R, so it may be that I am misunderstanding the terminology used to describe the descriptive and inferential analyses performed – but based on my disciplinary training (which I recognise may differ to those in this team), I would expect descriptives to be reported without the need for a GLM to be fitted – and then for the GLM to be fitted for any inferential analyses, e.g. to examine for any difference in outcome patterns between sub-groups.

o Please review this description of the descriptive and inferential analyses performed and edit as needed here (and in Results and Supplements) so that while it retains sufficient detail for replication, it is also clearer for the non-expert reader to understand exactly which tests have been performed, the rationale for these tests, and what data (Observed? Predicted?) are presented in Tables 2-4.

While there is information about ethical review and the informed consent process in the supplemental material, a summary of this information seems to have been omitted from the main text.

• Please can the authors provide summary information about ethical review, whether or not participants received incentives to participate, and consent process within the methods.

Results

The overall structure of the results is well-presented, with clear sub-headings that are coherent and logical to the research questions. However, I have concerns about the level of detail with which the analyses are currently presented, as it seems that there are both descriptive and inferential statistics that have not been reported in full.

Please can the authors address / reflect on the following:

• Descriptive statistics (percentages, mean scores) for all 4 outcomes should be reported in full – for both the overall sample and the sub-groups.

o Please report relevant data for overall sample for all 4 outcomes.

o Please report detailed data for the 2nd outcome (awareness of ABR) at both the overall and sub-group levels, e.g., as per Table 2 for awareness of antibiotics.

• As per my comments about the Methods – as currently drafted, there is ambiguity about whether the percentages and means presented in the main text and Tables 2-4 are observed data or predicted data.

o Please review language and reporting in both the Methods, Results and Supplements to be clear about the nature of these data.

• To my reading, Tables 2-4 only report descriptive statistics in relation to differences between sub-groups (i.e., percentage point differences). Further, I cannot see any results reported in relation to how the subdistrict variable is associated with the outcomes. I would normally expect to also see statistics reported related to any inferential tests of these differences (i.e., assuming hierarchical logistic regressions have been performed – which is

what appears to have been done based on current descriptions in methods and supplements), then associated p-values, odds ratios and confidence intervals for ORs should be reported – and potentially full details of models, such as betas and model fit statistics.

o Please report more details about the inferential tests (logistic regression models?) including relevant model fit statistics, statistical significance and effect size – and any hierarchical analysis performed in relation to the subdistrict variable

Discussion

As I am unsure about my interpretation of the statistical approach taken towards the data, I am reluctant to comment too deeply on the discussion. Without clear indication of the statistical significance and effect sizes, it is not clear whether the interpretations of the results are fully justified. If the manuscript is invited for revisions, then I would be willing to review and comment further on the discussion.

However, I do have the following comments that the authors may wish to reflect on:

• There is speculative discussion that education might be influencing / explaining associations (or lack) of between other independent variables and the outcome – without presenting any models that adjust for education, these claims should be made with caution and/or supported by evidence from the wider literature

• As this study reports cross-sectional data, claims about potential causal factors should be made with caution

• I find the 98% recruitment success rate to be notably high – while the authors have commented that this is useful for minimising selection bias / improving generalisability, I wonder whether there are other potential issues with this high recruitment success rate that the authors could reflect on, e.g., is there a potential risk of unintended coercion? E.g., there are potentially power differentials in relationships between researchers / healthcare staff and community members, that mean people may feel a social pressure to participate (or other undue influences). I am not suggesting this is the case in this study, but it is something I would encourage the authors to reflect on in their discussion e.g., what steps were taken to mitigate any risk of people not wanting to say no to recruiters, what steps were taken that helped contribute to high recruitment rates?

• There are some sections of the discussion that could benefit from reference to the wider literature about potential bias in these self-report survey study designs, such as where issues of social desirability are discussed in relation to potential measurement error with the questionnaires (e.g., lines 373-375 / 452-455) or misunderstanding the nature of the questions (e.g., 456-458)

Supplementary material

• Page 44 – typo in section 3.5.3 – refers to question 17 in 2.5.4.1 – I think this should be 3.5.4.1

• Suggestion for both knowledge outcome questionnaires (3.5.4) - for the avoidance of any doubt, in case these materials are used by other teams in future research, it would be helpful to indicate the 'correct' answer for each question, so that knowledge scores can be calculated in the same way – while most questions seem clear to me, there are a couple that I can imagine provoking a heated debate in a research or clinical meeting!

| | |
|---|---|
| **Reviewer** | **3** |
| **Name** | **Markham, Sarah** |
| **Affiliation** | **King's College, London, Biostatistics** |
| **Date** | **13-Sep-2025** |
| **COI** | **None** |

A competent and clinically relevant paper.

## VERSION 1 - AUTHOR RESPONSE

Reviewer: 1

Dr. Furqan Hashmi, University of the Punjab

Comments to the Author:

I have gone through the manuscript with interest that covers an important issue of AMR and antibiotic use, specially in the context of a LMIC.

Authors' response: Thank you very much for your time and careful consideration of our paper. We know how time-consuming peer-reviewing manuscripts can be.

Reviewer: 2

Dr. Sarah Golding , University of Surrey

Comments to the Author:

Manuscript Review - Associations between socio-demographic characteristics and knowledge about antibiotics, antibiotic resistance, and the use of antibiotics from a One Health perspective in rural Bangladesh: a descriptive cross-sectional study.

Authors' response: Thank you very much for your time and clearly very careful consideration of our paper. We know how time-consuming peer-reviewing manuscripts can be, and it is unfortunately increasingly uncommon to have a review that is as attentive as this one.

Overall comments

This manuscript reports a cross-sectional survey that examines – amongst the general adult population in rural communities in Bangladesh - their 1) awareness of antibiotics, 2) awareness of antibiotic resistance (ABR), 3) knowledge about use of antibiotics in humans and 4) knowledge about use of antibiotics in animals and in relation to the environment. The study has the potential to contribute to the literature – especially as it examines these issues from a One Health perspective - if some areas of the manuscript are sufficiently revised.

Overall, there is coherence to the manuscript – it is grounded in relevant literature, clearly written and easy for the reader to follow in most places, with a logical structure throughout. The manuscript offers originality by reporting on knowledge about appropriate use of antibiotics from a One Health perspective, within the context of rural communities in a lower-resource setting. The aims are clearly defined, with an appropriate study design and methods, and findings could have potential relevance for public health workers and researchers.

However, in its current draft, my recommendation is for major revisions, as I have some questions about how the statistical analyses are currently described and results presented. Within the Methods, there needs to be more clarity about the inferential analyses performed. Within the Results (or supplementary material), there needs to be additional descriptive and inferential statistics reported. I therefore cannot comment on the interpretations and conclusion at this stage. I have provided detailed comments below, which I think need to be addressed before the manuscript can be re-considered for publication.

Authors' response: We have addressed your detailed comments below where they require a response.

Abstract

Concisely written – clear overview of study methods and key results

Introduction

Clear and well-written – succinctly sets out the contextual threat from ABR, the context for this specific study, and the study aims/research questions. The authors are transparent about how these data have been collected, why they are being analysed for this study, and how data will be used in the future.

Please can the authors address the following, to help strengthen the rationale for this study:

• Provide a brief explanation about why there is a need to assess awareness and knowledge levels by socio-demographic variables (i.e., a sentence or two of theoretical justification, grounded in evidence from other studies in similar contexts, about why there is an expectation that knowledge might vary by age, education etc.)

Authors' response: This has now been added. See the second paragraph of the "Introduction" section.

Methods

For the most part, the methods have been reported sufficiently, and in line with two checklists appropriate for the study design. There is a mostly sufficient overview provided in the main text, with extensive additional detail provided in supplementary materials.

I am not an expert on cluster sampling methodology, so cannot comment on potential limitations of the approach adopted in this study – but I can follow from the authors' reporting the steps taken to create their sample, so I am satisfied this has been reported with sufficient transparency, supported by a reasoned argument.

However, I did find the description and rationale for the inferential analyses confusing in places - there seems to be some incoherence between the Methods and Results, regarding analyses described in the Methods, but results not reported (or only partially reported). I am also not clear exactly what inferential analyses have been performed, and in places I am not sure if the authors have reported actual (observed) data or the expected (predicted) data calculated from models.

Please can the authors address / reflect on the following:

• Lines 245-252: There is reference made to two types of analysis – one looking at outcomes (e.g., awareness, knowledge) for the overall target population (i.e. the whole dataset?) and one looking at outcomes by sub-group (e.g., age, education etc) – yet, I cannot see reported in the main text or the supplemental material the descriptive statistics for the whole dataset (e.g., Table 2 reports awareness of antibiotics for the sub-groups only, and I cannot see values for the outcome for the sample as a whole?).

o Please add these whole sample data for all outcomes.

Authors' response: Overall (i.e. whole sample) inferential results for the two awareness outcomes (awareness of antibiotics and awareness of antibiotic resistance) are provided under

the “Levels of awareness of antibiotics and antibiotic resistance” section. We did not provide overall inferential results for the two knowledge outcomes as we do not believe they are particularly informative as there is no obvious benchmark against which to compare them, given that we created the knowledge score ourselves (as there are no gold standard/universal tools available). Therefore, we believe they are only really useful when looking at relative knowledge levels among groups. However, we appreciate this comment and it is not for us to judge what readers may find useful or what may be useful in the future (e.g. if someone took our questionnaire and applied it). Therefore, we have now added the overall mean knowledge score values in a section called “Overall levels of correct and appropriate knowledge about antibiotics, ABR, and usage of antibiotics from a One Health perspective” (see 438 onwards). Therefore, overall/whole sample averages (and associated 95% CIs) are now provided for all outcomes.

• Lines 245-252: The text indicates that all models built included subdistrict as an independent variable – but I cannot see anywhere in the main text or supplemental materials any outputs that include values for the subdistrict variable.

o Please report the relevant descriptive and inferential statistics for associations between subdistrict and the 4 outcomes.

Authors' response: Subdistrict was only included to increase the precision of the focal estimates as the cluster sampling was stratified by subdistrict. It is widely accepted in the survey/sampling methodological literature that to maximise the benefit of any stratification in the sampling that your models should adjust for strata (e.g. see Korn and Graubard 1999 – Analysis of Health Surveys or any survey methods text book). We assumed the reason for the omission of the association results for subdistrict was therefore clear, as we did mention that we adjusted for subdistrict to increase precision, but we see that this is not necessarily clear, particularly for readers without awareness of the quantitative methodological issue. Therefore, we have now explicitly stated that we do not present these results as they were not relevant to the research questions (which were just looking at socio-demographic characteristics). See 360-365.

• Lines 252-262: I find this section of text confusing and hard to follow. It is not clear exactly what analyses and what data have been reported in the Results. For this type of study, I would expect to see descriptives reported from the observed data, (i.e., we asked 2160 people a question and X% answered correctly) for the whole sample, and for the sub-groups – and then a separate set of inferential analyses (e.g., logistic regressions) to examine sub-group differences. Yet, as currently drafted (and in combination with the Table 2-4 footnotes, and the additional information in the supplements), this section of text in the Methods suggests to me that the authors have not reported e.g., the % of e.g., Females/Males reporting awareness from the actual data (i.e., descriptive stats – percentages / means - based on observed data) but have instead reported predicted percentages/means (from a predictive model? To estimate percentages/means in the target population, extrapolated from the sample population?). I also cannot locate the results of inferential tests of difference in the results, such as p-values and effect sizes. I am not familiar with using R, so it may be that I am misunderstanding the

terminology used to describe the descriptive and inferential analyses performed – but based on my disciplinary training (which I recognise may differ to those in this team), I would expect descriptives to be reported without the need for a GLM to be fitted – and then for the GLM to be fitted for any inferential analyses, e.g. to examine for any difference in outcome patterns between sub-groups.

o Please review this description of the descriptive and inferential analyses performed and edit as needed here (and in Results and Supplements) so that while it retains sufficient detail for replication, it is also clearer for the non-expert reader to understand exactly which tests have been performed, the rationale for these tests, and what data (Observed? Predicted?) are presented in Tables 2-4.

Authors' response: We appreciate our description here could benefit from extensive revision to improve clarity, which we have done. Primarily, we have reworked it by starting simply by describing the types of results we produced via our analyses, and then we provide some technical details about the statistical approaches used to compute those results. We do not believe that our approach has any fundamental issues and that your main criticisms stem from a lack of understanding about our approach and its justifications, which we detail below. We have encountered similar confusion previously when trying to describe this increasingly popular, but still novel, approach. Therefore, we have also decided to cut out some of the technical, statistical detail provided previously in the main paper and just include it in the supplementary materials, to hopefully avoid confusion while still providing a comprehensive, technical explanation to interested readers. We now address each of your subpoints in detail:

1. Presentation of observed results. For the binary outcomes we do already provide the raw frequencies (i.e. counts), so you must have missed those in the text and table 2, and our percentage results (tables 2-4) do actually provide what you would see in the raw observed data if you computed those point estimates (i.e. %s and percentage point differences) by hand. However, we fully appreciate that the marginal effects approach, which uses model predictions to compute these %s and percentage point differences, can confuse unfamiliar readers and may sound like we are presenting model-predicted results that reflect hypothetical situations rather than the observed data. In reality, the results are based on model predictions but as they are based on predictions using the observed covariates the results are what you would get if you computed them by hand from the observed data. The reason for taking this seemingly over complicated marginal effects approach is that it allows us a consistent way to compute differences between subgroups on the percentage point scale, rather than as the more common odds ratios, along with the associated 95% confidence intervals, in the context of adjusting the standard errors for the clustered sampling design, because there are no obvious alternative approaches that allow this. We much prefer percentage point differences as they are far more intuitive and easily interpretable from a public health perspective (there are lots of papers that argue for this, e.g. see the JAMA paper from 2020 "Estimating Risk Ratios and Risk Differences Alternatives to Odds Ratios"). We have now explained this explicitly (and included the reference above) in the methods so these points (that the results reflect the observed data and why we took this approach) is hopefully clearer to a reader. See lines 340-344.
2. Lack of inferential results, p-values, effects sizes, and an expectation of descriptives without the need for a GLM. We would respectfully argue that this part of the comment

appears to follow from a misunderstanding both about our approach and certain statistical concepts.

a. First, we are explicitly taking a descriptive research approach to this study, i.e. we are seeking to describe characteristics and associations as they exist in our target populations, as opposed to doing causal inference (estimating causal effects) or prediction (estimating predictors of some outcome). To further clarify and justify this approach we have slightly reworked part of the introduction (see lines 141-157) and added a key supporting reference for this approach here. However, we would argue it was already explicitly stated/justified in the title, introduction and in the "Approach and reporting" section (along with a key reference), and we would also argue the "Statistical analyses" section were already consistent with this approach. However, we fully accept the "Statistical analyses" section lacked clarity and was overly technical for a non-specialist (and as per our response to your earlier comment we have reworked it accordingly).
b. Second, we provide what are commonly called descriptive statistics in table 1, i.e. summary measures of key characteristics for the sample. However, we would argue that a better term is summary statistic (as we use) or sample statistic, as in our experience non-methodologists are often confused about the distinction between descriptive statistics and inferential statistics, as is maybe the case here. These descriptive or summary statistics are just point estimates and only apply to the sample. They are not intended to be used to infer anything about any target population, nor should they be (this would be a fundamental statistical error as there is no measure of uncertainty associated with them). Our inferential results however are all intended to allow readers to draw conclusions about the target populations, via our inferential measures, which we would argue we do already explicitly state are 95% CIs, but we have slightly modified this section for further clarity (see lines 388-389). Therefore, as we include 95% CIs for all %s and percentage point differences in tables 2-4 those tables are all inferential results tables, as the 95% CIs provide a means of inference to the target population. Therefore, just because we haven't included any statistical tests or p-values does not mean we have not included inferential results. This appears to be a misunderstanding about what constitutes an inferential result. Although p-values and 95% CIs are the most common inferential measures there are many others, so it would be wrong to imply there are no inferential measures. We have not included any tests or p-values because we are not testing anything. Again, this appears to stem from a misunderstanding about our approach and the appropriate usage of hypothesis testing. We are estimating summary measures of outcomes (in terms of percentages) for the overall population and subgroups and measures of association between our outcomes and variation across subgroups. That only requires suitable summary and associational measures and associated 95% CIs to make inferences about the likely value of those summary and associational measures in the target population. P-values have also been widely and repeatedly criticised, which is another reason why we avoid including any, but primarily they just don't add anything that our 95% CIs already tell us and in a much more nuanced and useful way, and they are not required or useful in this framework. Going back to point a, you also talk about a lack of effect sizes but an effect size is just a

measure of association that is meant to reflect a causal effect. Therefore, as we are not doing causal inference here we do not include any measures of effect. Again, this seems to reflect a misunderstanding about our approach and when effect sizes are appropriate. However, we do include measures of association, which are the descriptive equivalent: our percentage point differences (i.e. a percentage point difference could be an effect size, just like an odds ratio, if it reflected a causal effect, say from a robust RCT). Therefore, overall we hope that we have clarified that our approach is both consistent with our research goals and the statistical analyses are consistent with those goals. We would also highlight that our approach and statistical analyses are supported by methodological guidance and recommendations in the literature for these types of studies, which we reference in relevant places in the "Inferential analyses" section.

c. On a final more general note, as this comment appears to cover all the major issues you had with our paper, if you do continue to disagree with anything to do with our basic approach and analyses we respectfully request that you please provide evidence-based justification (i.e. references) for those disagreements and why any changes should be undertaken, as we believe our approaches are justified and grounded in guidance and recommendations published by methodological experts, which we provide. The most relevant references that justify our approach would be 1) "A Framework for Descriptive Epidemiology" (https://pmc.ncbi.nlm.nih.gov/articles/PMC10144679/), 2) "On the Need to Revitalize Descriptive Epidemiology" (https://academic.oup.com/aje/article/191/7/1174/6552325), 3) "A Primer on Marginal Effects" (https://link.springer.com/article/10.1007/s40273-014-0224-0). Also, we didn't cite this but you may also find "Data Science is science's second chance to get causal inference right" (https://arxiv.org/abs/1804.10846), which covers the distinction between description, causal inference, and prediction in quantitative research. We do not think it would be appropriate or fair to require us to amend our approach or methods just because you are not familiar with certain terminology, your training hasn't covered these approaches, or you would expect different results just because they are not what you are used to etc. Finally, on the point of terminology, we would also note that none of the terminology we use is related to R, as you questioned. It is general statistical terminology related to these approaches.

While there is information about ethical review and the informed consent process in the supplemental material, a summary of this information seems to have been omitted from the main text.

• Please can the authors provide summary information about ethical review, whether or not participants received incentives to participate, and consent process within the methods.

Authors' response: Please see the "Ethics approval" section near the end of the manuscript (after the "Conclusion" section). We assume you missed this because it covers all these issues. This is also the required location for this information as per BMJ Open so we will not move it to the "Methods" section.

Results

The overall structure of the results is well-presented, with clear sub-headings that are coherent and logical to the research questions. However, I have concerns about the level of detail with which the analyses are currently presented, as it seems that there are both descriptive and inferential statistics that have not been reported in full.

Please can the authors address / reflect on the following:

• Descriptive statistics (percentages, mean scores) for all 4 outcomes should be reported in full – for both the overall sample and the sub-groups.

Authors' response: Please see our response to your earlier comment above where you asked for the same thing, which we have clarified and provided.

o Please report relevant data for overall sample for all 4 outcomes.

Authors' response: As per the above bullet point.

o Please report detailed data for the 2nd outcome (awareness of ABR) at both the overall and sub-group levels, e.g., as per Table 2 for awareness of antibiotics.

Authors' response: This was an intentional decision as we did not believe that adding these subgroup results would add much in addition to the subgroup results for the self-reported antibiotic awareness outcome as they are very similar outcomes. We realise that we did not explicitly state this however. We have therefore now clarified this decision. See lines 148-149.

• As per my comments about the Methods – as currently drafted, there is ambiguity about whether the percentages and means presented in the main text and Tables 2-4 are observed data or predicted data.

Authors' response: Please see our response to your earlier, large comment above relating to the same issue, where we believe we have addressed this issue now.

o Please review language and reporting in both the Methods, Results and Supplements to be clear about the nature of these data.

Authors' response: Again, see our response to your earlier comment above relating to the same issue, where we believe we have addressed this issue now given how we have reworked our description of the methods.

• To my reading, Tables 2-4 only report descriptive statistics in relation to differences between sub-groups (i.e., percentage point differences). Further, I cannot see any results reported in relation to how the subdistrict variable is associated with the outcomes. I would normally expect to also see statistics reported related to any inferential tests of these differences (i.e., assuming hierarchical logistic regressions have been performed – which is what appears to have been done based on current descriptions in methods and supplements), then associated p-values, odds ratios and confidence intervals for ORs should be reported – and potentially full details of models, such as betas and model fit statistics.

Authors' response: Again, we would respectfully argue that this comment appears to follow from a misunderstanding about what constitutes a descriptive statistic/result vs an inferential one and about our marginal effects based approach to estimation. As we explained earlier, we provide 95% CIs for all our measures of association, i.e. the percentage point differences, and so these are not descriptive statistics: they allow you to infer what the likely corresponding percentage point difference is in the relevant target population. Hence, they are inferential results. Also as described earlier, we do not present any p-values as we are not testing any hypotheses. And as touched on earlier, although we use logistic regression models we compute our results via marginal effects methods primarily to allow us to avoid having to present our measures of association for our binary/binomial outcomes as odds ratios, which make for less intuitive and easy interpretation compared to absolute-scale measures like percentage point differences (we have added a reference to support this, see reference on line 344). Indeed, we would argue that the almost universal presentation of odds ratios in survey analyses of binary outcomes is a major limitation that could be easily avoided by using approaches like we do here. Therefore, the model coefficients are not relevant as those relate to logit-scale associations, but we use the models to instead get the more useful percentage and absolute-scale measures of association. See below for the comment on model fit statistics. Therefore, we again respectfully argue that what you would normally expect to see is not a fair or valid basis for critiquing our results when it's not backed up by any authoritative references, as there are many common practices in the literature (across disciplines) that are heavily criticised by methodologists (often for decades). Again, we feel we have provided sufficient justification for our approaches and would argue any counter arguments against them must be justified based on authoritative evidence/references.

o Please report more details about the inferential tests (logistic regression models?) including relevant model fit statistics, statistical significance and effect size – and any hierarchical analysis performed in relation to the subdistrict variable

Authors' response: Again, we would respectfully argue that this comment appears to follow from a misunderstanding about our approach and certain statistical issues following from that.

In terms of inferential tests, statistical significance and effect sizes we would just refer to our previous responses on this issue, namely that we do provide suitable inferential results already but that our approach does not involve hypothesis testing. In terms of model fit statistics these are not relevant for two reasons. First, we are not seeking to find a "best model" as you would do with, e.g., prediction modelling, based on model fit. There is only one appropriate model per outcome and covariate of interest combination, and that is the model for that outcome that contains the covariate of interest plus the sampling strata. There is no model selection possible in this approach. Again, this relates to the distinct approaches to description, causal inference and prediction. We are just using the models, along with marginal effects methods, as a way to estimate summary measures of our outcomes and associations (i.e. percentages and percentage point differences), with their associated 95% CIs allowing us to make statistical inferences about these measures to the target populations. Again, although this may seem overly complicated there are not actually any obvious alternatives when you want to estimate percentage point differences (or risk differences) while also adjusting your variances for strata and clustered sampling (maybe a complicated bootstrapping approach of such estimates when computed by hand, but that is computationally intensive). So our approach is really just a means to an end that unfortunately does seem to create a lot of confusion for what is ultimately a very simple end goal. Second, certain model fit statistics can indicate issues with model assumptions, and for our binomial GLMs for the knowledge scores it is true that the models could suffer from overdispersion due to violations of the assumption that the binomial trials are not independent. However, we adjust for clustering at the highest level (the sampling clusters), which has been shown to correctly adjust for lower-level clustering. We have however added a reference justifying this as we did not explicitly address this before (see lines 384-388 and the reference included). Therefore, there is no reason for us to include model fit statistics as they are not relevant for our approach and are not involved/used in it.

Discussion

As I am unsure about my interpretation of the statistical approach taken towards the data, I am reluctant to comment too deeply on the discussion. Without clear indication of the statistical significance and effect sizes, it is not clear whether the interpretations of the results are fully justified. If the manuscript is invited for revisions, then I would be willing to review and comment further on the discussion.

Authors' response: Thank you. We would of course hope that you are able to review in light of our responses, which have taken a long time to prepare, but contingent on having a broad understanding of our approach and the fact that this does not involve hypothesis testing or causal inference, and that you should therefore not expect to see "statistical significance" or effect sizes. As we focus solely on confidence intervals if you want to review their interpretation we would recommend this concise guide: [https://saspaper4.mcg-ct.com/Scientists_rise_up_against_statistical_significance.pdf](https://saspaper4.mcg-ct.com/Scientists_rise_up_against_statistical_significance.pdf) Although it is implicitly written from the perspective of doing causal inference rather than descriptive inference (and so talks about effect sizes, but the principles are the same).

However, I do have the following comments that the authors may wish to reflect on:

• There is speculative discussion that education might be influencing / explaining associations (or lack) of between other independent variables and the outcome – without presenting any models that adjust for education, these claims should be made with caution and/or supported by evidence from the wider literature

Authors' response: As previously described our goals were descriptive, so while you are correct that we could explore this issue further in our data with more causal-focused analyses as it's not at all the focus of the study and a post-hoc finding we will leave it as discussion/speculation. However, we have amended our discussion of these issues to be more explicit that they are speculative and should be treated cautiously as such. In particular, see lines 522-527, 536-538, and 557-561.

• As this study reports cross-sectional data, claims about potential causal factors should be made with caution

Authors' response: We do not believe we are making any strong causal claims, particularly following the edits based on your comment above, and obviously as discussed this is explicitly primarily a descriptive study so we are well aware of the distinction and limitations/alternative focus of this type of study. As you say above though, we do provide some speculation about possible underlying causal processes, which clearly may still be worth considering in a descriptive study, but we do not believe that we imply anywhere that these are strong claims that we are willing to stand behind (again, particularly after the edits following your comment above), rather than simply "food for thought". Readers are free to agree or disagree with our speculations where we touch on potential underlying causal processes and we are certainly not tied to any of the speculations we make, but we do believe our descriptive results are quite robust for the strengths outlined. If you think we need to make any further changes it would really help if you were explicit and listed all areas where you feel we have overstepped in some way and we are willing to revisit this.

• I find the 98% recruitment success rate to be notably high – while the authors have commented that this is useful for minimising selection bias / improving generalisability, I wonder whether there are other potential issues with this high recruitment success rate that the authors could reflect on, e.g., is there a potential risk of unintended coercion? E.g., there are potentially power differentials in relationships between researchers / healthcare staff and community members, that mean people may feel a social pressure to participate (or other undue influences). I am not suggesting this is the case in this study, but it is something I would encourage the authors to reflect on in their discussion e.g., what steps were taken to mitigate any risk of people not wanting to say no to recruiters, what steps were taken that helped contribute to high recruitment rates?

Authors' response: We assume that you are maybe not familiar with conducting research in LMICs? In our experience across many Asian and African LMIC settings responses rates are

always very high, without any incentives. We believe that the ethics section already covers these issues, as it explicitly states we offered no incentives and that all participants were fully informed of the study and their right to participate or not. We took no steps to increase recruitment and our previous extensive experience working in these settings is that there is absolutely no need to. People are almost always willing to participate in a brief survey like this. This is one of the major positives or working in LMICs compared to HICs, where people are generally unwilling to participate. As the study received ethical approval from both the UK and in-country ethics committees we don't think that we need to add anything further on this, as it's a very typical outcome for research in LMICs. However, if you feel strongly against this view we are certainly willing to revisit this issue.

• There are some sections of the discussion that could benefit from reference to the wider literature about potential bias in these self-report survey study designs, such as where issues of social desirability are discussed in relation to potential measurement error with the questionnaires (e.g., lines 373-375 / 452-455) or misunderstanding the nature of the questions (e.g., 456-458)

Authors' response: We have slightly amended the identified sentences and added a key supporting reference (see lines 492-496 and 576-583).

Supplementary material

• Page 44 – typo in section 3.5.3 – refers to question 17 in 2.5.4.1 – I think this should be 3.5.4.1

Authors' response: Thank you. Corrected.

• Suggestion for both knowledge outcome questionnaires (3.5.4) - for the avoidance of any doubt, in case these materials are used by other teams in future research, it would be helpful to indicate the 'correct' answer for each question, so that knowledge scores can be calculated in the same way – while most questions seem clear to me, there are a couple that I can imagine provoking a heated debate in a research or clinical meeting!

Authors' response: Thank you for the suggestion. This has been amended accordingly.

Reviewer: 3

Dr. Sarah Markham, King's College, London, Birkbeck University of London

Comments to the Author:

A competent and clinically relevant paper.

Authors' response: Thank you very much for your time and careful consideration of our paper. We know how time-consuming peer-reviewing manuscripts can be.

Reviewer: 1

If you have selected 'Yes' above, please provide details of any competing interests.: Not applicable

Reviewer: 2

If you have selected 'Yes' above, please provide details of any competing interests.: Not applicable.

Reviewer: 3

If you have selected 'Yes' above, please provide details of any competing interests.: Not applicable

## VERSION 2 - REVIEW

| | |
|---|---|
| **Reviewer** | **2** |
| **Name** | **Golding , Sarah** |
| **Affiliation** | **University of Surrey, School of Psychology** |
| **Date** | **14-Nov-2025** |
| **COI** | |

Manuscript Review (Revised version R1) - Associations between socio-demographic characteristics and knowledge about antibiotics, antibiotic resistance, and the use of antibiotics from a One Health perspective in rural Bangladesh: a descriptive cross-sectional study.

Overall Comments

I would like to thank the authors for their detailed responses to my comments made in the previous round of peer review and for making the revisions to the manuscript in response to my comments. I appreciate how time-consuming the peer review process can be, and I am very grateful for the detailed and robust responses provided by the authors, particularly in

relation to my confusion about the statistics that have been reported – thank you for taking the time to clarify your methodological approach. I am happy to recommend publication.

Rather than provide full point-by-point responses, I'll summarise as follows;

• The minor edits made throughout the manuscript are all sufficient (e.g., additions and edits to introduction, discussion and results) – thank you for addressing these points, and I am satisfied with your arguments where you have chosen not to make edits

• Ethics statement – yes, I had seen this section, but had not realised the journal requirement was for it to be placed in this location outside the main text, my apologies

• Discussion – I have re-read this following the edits made and the clarification of the methodological approach by the authors. I am satisfied that the discussion reflects a fair interpretation of the results presented and it suitably relates the findings to the wider literature.

Regarding the methods section and methodological approach. Yes, I had essentially been confused by the approach taken and how it had been reported - and I wasn't clear whether it was my misunderstanding or whether more information was required:

• As you outline in your 'presentation of observed results' response – I had been confused by the notion that the percentages reported were based on model predictions, which made me question my understanding of the exact nature of the aims of the study and the analysis (i.e., was it purely descriptive as set out in the introduction or was there some hypothesis testing that had not been fully set out?). Related to this, because (as you point out in one of your later responses) confidence intervals allow for statistical inference, this led to further doubt in my interpretations of the results and the statistical analyses presented. In short, I wasn't sure whether I was looking at the outputs of analyses that were reporting description/association only and / or the (partial) outputs of predictive or causal analyses.

• I fully agree with your later point, that it would be entirely unreasonable to ask for the methods / approach to be changed, simply because of the lack of understanding or lack of familiarity with a methodological approach on the part of a reviewer. I had requested the additional statistics (e.g., p-values, effect sizes, model fit, etc) to be reported, as my confusion about how logistic regressions were being used in this study (to calculate the %age point difference, as an alternative to odds ratios), meant I had thought a causal or predictive analysis had been performed, and therefore felt these other statistics were missing from the reporting. Based on your helpful responses about why you adopted this approach and the clarification about the study being descriptive and not being about hypothesis testing, I am satisfied these other statistics are not required/appropriate for this study.

• Thank you for reworking the methods section of manuscript. It is now much clearer to follow the steps taken and the rationale. Thank you also for further outlining the rationale

for adopting the marginal effects approach in your responses, and for the additional references. All of this effort is much appreciated.
